# Improving child mental health and learning outcomes and reducing stigma and discrimination in conflict setting: findings from a cluster randomized controlled trial of a classroom‐based psychosocial intervention in rural primary schools in Afghanistan

**DOI:** 10.1111/jcpp.70125

**Published:** 2026-01-25

**Authors:** Jean‐Francois Trani, Yiqi Zhu, Saria Bechara, Shuya Yin, Parul Bakhshi, Ian Kaplan, Ramkrishna K. Singh, Mohammed A. Modaber, Hashim Rawab, Madelyn Yoo, Kim Thuy Seelinger, Ganesh M. Babulal, Ramesh Raghavan

**Affiliations:** ^1^ School of Public Health Washington University in St. Louis St. Louis MO USA; ^2^ Department of Psychology University of Johannesburg Johannesburg South Africa; ^3^ National Conservatory of Arts and Crafts, Interdisciplinary Laboratory for Economic Sociology Paris France; ^4^ Department of Neurology Washington University School of Medicine St. Louis MO USA; ^5^ Adelphi University New York USA; ^6^ Occupational Therapy Washington University School of Medicine St. Louis MO USA; ^7^ Norwegian Afghanistan Committee Kabul Afghanistan; ^8^ Division of Population Health and Aplied Health Science, Faculty of Medicine Memorial University of Newfoundland St. John's, NL Canada; ^9^ Silver School of Social Work New York University New York NY USA

**Keywords:** Afghanistan, children mental health, randomized controlled trial, classroom‐based psychosocial intervention

## Abstract

**Background:**

Conflict and crises have long‐lasting and dramatic consequences on the mental health of children. We aimed to investigate the effectiveness of a psychosocial intervention on child mental health in Afghanistan.

**Methods:**

A two‐arm cluster‐randomized controlled trial was conducted in 83 rural primary schools within three provinces of Afghanistan. Children in Grades 3–6, their teachers, and one adult family member were enrolled. Schools were randomly assigned (1:1) to one of two groups: a treatment group composed of entire classes receiving a week‐long classroom‐based teacher‐and‐child psychosocial training, a one‐day family engagement component, and a community‐based system dynamics workshop; and a control group. Primary outcomes were anxiety, depression, life skills, self‐efficacy, and resilience of the child. Secondary outcomes included reading and mathematical literacy, mathematical problem‐solving, and school‐based discrimination and stigma. This trial is registered with the International Standard Randomized Controlled Trials Number registry (ISRCTN83632872).

**Results:**

In June 2021, 40 schools and *n* = 2,262 children were randomly assigned to the intervention group and 43 schools and *n* = 2,277 children to the control group. Preintervention survey started October 2, 2021 (first batch) and April 10^th^, 2023 (second batch). After a minimum of 4‐month intervention, a postintervention survey took place. No treatment effects were found on anxiety, depression, resilience, self‐efficacy, life skills, or stigma. Effects were found for academic outcomes and school‐based discrimination. Shorter interventions displayed reductions in depression, anxiety, stigma, and discrimination, and an increase in life skills. Additional analyses showed significant effects on several outcomes for boys, on a few outcomes for girls, and in areas where the governmental did not disrupt the process.

**Conclusions:**

Classroom‐based interventions delivered by trained field‐based educational staff can effectively promote child mental health, social–emotional skills, and academic outcomes, and reduce stigma and discrimination among subgroups of children in conflict and crisis settings and have viable potential for scalability.

## Introduction

The global burden of mental health disorders has been steadily increasing worldwide since 1990, and major mental disorders are leading causes of disease burden and disability, especially among children (GBD 2019 Mental Disorders Collaborators, 2022). This burden is particularly acute within low‐ and middle‐income countries (LMICs), with their predominantly younger child and youth populations that are at higher risk of poor mental health outcomes (McGorry et al., 2024). The situation is even more dire for generations of children in Afghanistan since 1979, who have grown up being exposed to harm, oppression, violence, displacement, human rights violations, inequities, and pervasive poverty (Nascimento et al., 2022). This long‐lasting, multipronged crisis has had dramatic consequences on the mental health of its population (Alemi et al., 2018, 2023; Kovess‐Masfety, Keyes, Karam, Sabawoon, & Sarwari, 2021). Afghan children have been particularly impacted by the trauma of war and political upheaval, experiencing the loss of family, friends, and community members, witnessing violence, risking injury or death, and daily social and economic stressors resulting from poverty, poor health, malnutrition, domestic and family violence, insecurity, social isolation, and limited access to healthcare and education (Nascimento et al., 2022; Panter‐Brick, Eggerman, Gonzalez, & Safdar, 2009; Trani & Bakhshi, 2013). These stressors are important social determinants of poor mental health outcomes, particularly anxiety, depression, posttraumatic stress, and poor functioning (Catani et al., 2009; Dyer et al., 2009; Panter‐Brick et al., 2009).

Children in Afghanistan grow up amid persistent conflict, poverty, and restrictive social norms that profoundly shape their education, mental health, and overall development (Panter‐Brick, Grimon, Kalin, & Eggerman, 2015). Exposure to conflict‐related violence, domestic violence, and peer victimization contributes to cumulative trauma, while food insecurity and child labor limit school attendance and learning opportunities. Gender and cultural barriers – exacerbated by the Taliban's restrictions on girls' education – further curtail access, particularly for girls and children with disabilities, who face entrenched stigma and exclusion (Trani et al., 2024). Even when children attend school, they encounter overcrowded classrooms, poorly trained teachers, inadequate infrastructure, and curricula increasingly shaped by ideological agendas that reduce the scope of secular subjects (Amiri & Jackson, 2021).

These intersecting adversities disrupt family and community support networks, reinforce mental health stigma, and undermine psychosocial well‐being, emphasizing the need for interventions addressing both the individual and structural determinants of child resilience and development in Afghanistan (Corboz, Siddiq, Hemat, Chirwa, & Jewkes, 2019; Trani et al., 2019). In this context, children face daily stressors rooted in decades of insecurity and socioeconomic hardship (Alemi et al., 2023; Cameron et al., 2021; Trani et al., 2024; Trani, Biggeri, & Mauro, 2013). To thrive despite these challenges, children need support to build resilience, cope with trauma, adapt, and develop a sense of hope (Ventevogel, Jordans, Eggerman, van Mierlo, & Panter‐Brick, 2013).

Given these realities, interventions must adopt a socioecological perspective, recognizing children – with their individual coping capacity, beliefs and values, intelligence, and creativity – as embedded within interconnected family, school, and community (peers, village members) microsystems shaped by cultural and religious norms and practices, political instability, and economic constraints (Bronfenbrenner, 1979; Panter‐Brick & Eggerman, 2011; Tol, Jordans, Kohrt, Betancourt, & Komproe, 2013; Trani & Bakhshi, 2013; Ventevogel et al., 2013). School‐based interventions are particularly promising because they can address barriers across multiple ecological levels – from the immediate microsystem of home and classroom to the macrosystem of gender norms, governance constraints, and the chronosystem of environmental and sociopolitical changes (Dieu Yin, Low, & Mishu, 2025). In Afghanistan, chronic poverty, shortages of trained teachers (particularly female teachers), limited school infrastructure, and entrenched gender norms intersect with mesosystem‐level gaps in school–family collaboration, exosystem governance constraints, and chronosystem shocks such as conflict, regime change, and the coronavirus disease 2019 (COVID‐19) pandemic. For many Afghan children, schools remain one of the few relatively safe, protective, and supportive environments for cognitive, social, and emotional development across these bioecological systems (Betancourt et al., 2013a; Burde & Linden, 2013; Trani et al., 2019; Winthrop & Kirk, 2008).

While a socioecological approach to child mental health and resilience in Afghanistan would arguably require integrating specialized mental health services into general healthcare, the existing healthcare system lacks the capacity to provide such support (Ventevogel et al., 2013). Barriers to accessing mental healthcare services in LMICs are substantial, and even more so in a conflict and crisis context like Afghanistan. There is limited availability of specialized service providers in LMICs (Whitley, 2015). Afghanistan had an estimated 100 psychiatrists in 2019 (Alemi et al., 2023). In Afghanistan, other barriers to accessing care include medication and travel costs, private doctor fees, large distances, and a lack of safety on the way to seeking care. Furthermore, mental illness (e.g. depression and psychosis) is highly stigmatized in Afghan society, particularly regarding work and marriage (Nine, Najm, Allan, & Gronholm, 2022). Therefore, individuals with mental disorders prefer to keep their suffering to themselves or within the family to avoid mockery, blaming, labelling, and the resulting feeling of shame for themselves and their caregivers (Nine et al., 2022; Oriya & Alekozai, 2022). The fact that many existing mental health interventions suffer from cultural insensitivity represents yet another barrier to seeking help, as does limited engagement with local healthcare professionals (Hamza & Hicks, 2021; Seidi & Jaff, 2019).

In the absence of formal healthcare services, community‐based psychosocial interventions that address socioecological determinants of poor mental health have been increasingly viewed as the only viable alternative for addressing poor mental health in crisis contexts (Heap et al., 2022). The current evidence base for psychosocial interventions' effectiveness and mechanisms of change for war‐affected children is limited and lacks rigorous research methods (O'Sullivan, Bosqui, & Shannon, 2016). This gap underscores the critical role of school‐based interventions, which can offer accessible, nonstigmatizing platforms for promoting psychosocial well‐being and resilience among children in conflict‐affected settings. Some evidence exists that school‐based interventions may positively impact symptoms of anxiety, distress, or depression while improving resilience through building self‐care, self‐esteem, and supportive relationships. For example, a randomized controlled trial conducted in schools in Nepal showed increased prosocial attitudes among girls and lower distress and aggression among boys (Jordans et al., 2010). Good peer relationships have also been shown to improve children's mental health in conflict settings (Paardekooper, De Jong, & Hermanns, 1999).

Evidence from high‐income countries links social and emotional learning (SEL) interventions to improved academic achievement, well‐being, and social–emotional skills (Smithers et al., 2018; Wigelsworth et al., 2020). Emerging studies in low‐ and middle‐income and crisis settings show similar promise (Deitz, Lahmann, & Thompson, 2021; Kim et al., 2023) yet remain too limited to isolate active components or explain impact heterogeneity (Durlak, Mahoney, & Boyle, 2022; Durlak, Weissberg, Dymnicki, Taylor, & Schellinger, 2011; Taylor, Oberle, Durlak, & Weissberg, 2017). Recent evaluations in conflict contexts report gains in academic outcomes and school safety but modest effects on social–emotional skills (Aber, Dolan, Kim, & Brown, 2021; Torrente et al., 2019), highlighting the need to identify the most effective intervention strategies.

The present study's contribution is to evaluate the effectiveness of a culturally adapted, two‐component SEL intervention for children of primary school age in rural Afghanistan. The intervention was implemented by locally recruited and trained field‐based educational staff. Primary (proximal) outcomes included depression and anxiety symptoms, while secondary (distal) outcomes encompassed noncognitive learning skills – life skills, self‐efficacy, resilience – academic achievement in reading and mathematics, and improvements in school and village climate measured through reduced stigma and discrimination.

The primary research question examined whether a socioecologically informed SEL intervention could enhance children's mental well‐being, learning outcomes, and perceptions of school and community climate. The first hypothesis posited that children receiving the intervention would exhibit greater reductions in anxiety and depression, improved life skills, higher self‐efficacy and resilience, stronger academic performance, and lower stigma and discrimination compared to controls. Given prior evidence suggesting school‐based interventions in conflict‐affected settings may benefit girls disproportionately (Ager et al., 2011; Jordans et al., 2010; Tol et al., 2008)– although Jordans et al. (2010) showed the intervention was more beneficial for girls only on prosocial behaviors, while it was more beneficial for boys on psychological difficulties and aggression– the second research question explored gender differences in treatment effects, with the second hypothesis predicting greater benefits for girls than boys. The third research question explored area‐level differences in treatment effects, with the third hypothesis predicting greater benefits in areas with higher implementation intensity, due to stronger schools' community engagement and a more conducive environment.

## Methods

### Study design

We conducted a two‐stage cluster‐randomized controlled trial in rural primary schools in three provinces of Afghanistan, namely, Badakhshan, Ghazni, and Takhar, between May 1, 2021, and December 31, 2023, in partnership with two nongovernmental organizations (NGOs), the Norwegian Afghanistan Committee and the Swedish Committee for Afghanistan. Badakhshan is located in the northeastern part of the country and shares a border with Tajikistan to the north and east, Pakistan to the southeast, and China through a narrow corridor called the Wakhan Corridor. It is bordered by Takhar, situated in the northeast of the country, next to Tajikistan. Ghazni is situated in the southeastern part of the country. Badakhshan is the largest and poorest of the three provinces, with most of its territory occupied by the Hindu Kush and Pamir Mountain ranges. The populations of these three rural provinces are composed of farmers and herders. Mining activities are essential in both Badakhshan (lapis lazuli and, recently, other gemstones) and Takhar (gold, coal, salt, and stones for construction). The various ethnic groups of Afghanistan are represented in the three provinces: the two main ethnic groups – Pashto and Tajik – and several minorities – Hazara, Uzbek, Kyrgyz, Turkmen, and Balochi. Sporadic resistance to the Taliban regime has been recorded in all three provinces.

### Randomization and masking

**Figure 1:**
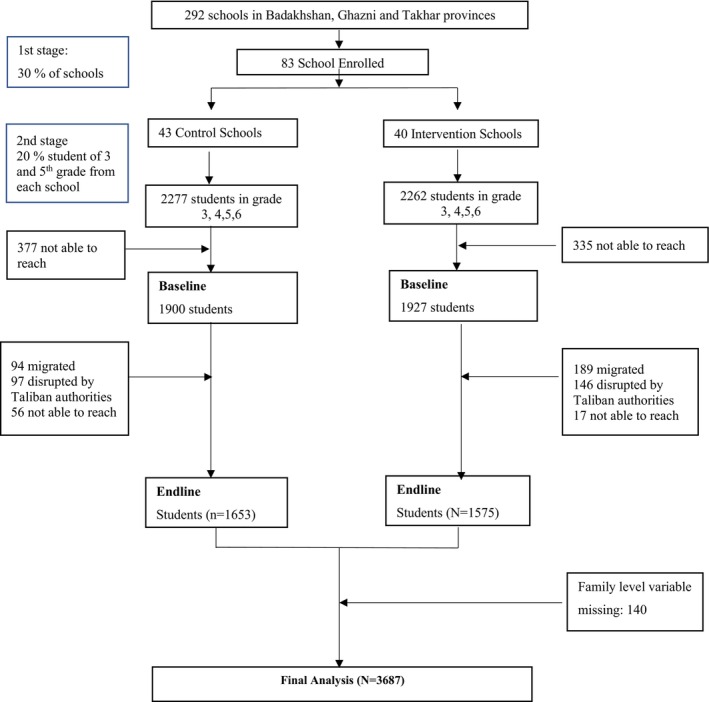
Trial profile

The authors first enumerated all primary schools in each province, whether governmental or community‐based, that received support from the two international NGOs and had at least one classroom for Grades 3–5. Figure 1 displays the CONSORT‐style diagram for this study. From among a total of 292 schools across these three provinces, we used a random number generator to select schools within each province (a total of 83 schools). To avoid contamination, only one school was randomly selected in each village (where more than one school existed). The sample was stratified by province, and schools within each province were randomly assigned in a 1:1 ratio to one of two groups (40 to the intervention group and the remaining to the control group).

Within each school, we selected the entire Grade 3–5 class when the class size was below 20 children. In classes with more than 20 children, we randomly selected 20 children as participants. From a total cohort of 3,254 children, we enrolled 1,522 children in the intervention group and 1732 in the control group. Children, their teacher (one per class), and their parents formed this study's participants. The intervention group received the culturally adapted participatory psychosocial intervention (described below), while the control group received no intervention and continued with the usual teaching and learning processes. All participants provided written (or witnessed, if they were unable to write) informed consent.

The study was approved by the Ministry of Education in Afghanistan and Washington University in St. Louis. The trial is registered with the International Standard Randomized Controlled Trials Number registry (ISRCTN83632872).

### Intervention

A two‐component intervention was delivered to the children, their teacher, and their parents. It consisted of (a) a week‐long classroom‐based training with teachers and children together; (b) a 1‐day training with parents (see Appendix S1). Both components used a culturally adapted version of the ‘A hopeful, healthy, and happy living and learning toolkit’ developed by the International Federation of the Red Cross and Red Crescent Societies in response to the COVID‐19 pandemic (REPSSI & IFRC Reference Centre for Psychosocial Support, 2021; see Appendix S2).

The toolkit has a multipronged theoretical foundation that integrates evidence‐based frameworks with context‐specific adaptations to promote child mental health and well‐being. First, teacher–student and parent components are grounded in the SEL framework, which has demonstrated beneficial effects on child mental health, reductions in risk behaviors, and improvements in social, emotional, behavioral, and academic outcomes in school settings (Durlak et al., 2011, 2022; Payton et al., 2000). The teacher–student component targets core SEL competencies and attitudes and incorporates mindfulness‐based strategies – including yoga as a culturally relevant practice – to strengthen self‐awareness and self‐regulation (Bockmann & Yu, 2023). The parent component draws on evidence for positive parenting and reduced use of punitive discipline, addressing behavioral norms prevalent in low‐resource and conflict‐affected settings, including Afghanistan (Knerr, Gardner, & Cluver, 2013).

Second, gray literature indicates that the toolkit has enhanced students' cognitive, social, and emotional skills in Greece and Slovakia, with teachers reporting improved classroom dynamics and emotional regulation (Bempi et al., 2024). These experiences highlight the importance of cultural adaptation, contextual awareness, and sustained stakeholder engagement, including collaboration with teachers and school principals. Early engagement with educational authorities, rigorous teacher training, flexible scheduling, and continuous monitoring and feedback were identified as key to maintaining fidelity while allowing timely adaptations.

Third, research highlights that SEL interventions can support mental health, resilience, and psychosocial well‐being in conflict settings (Bosqui & Marshoud, 2018). Socioecological approaches that engage children across multiple systems – home, school, peers, and community – and address interactions between these systems have been shown to foster resilience and mental health (Tol, Haroz, Hock, Kane, & Jordans, 2014). Studies from Afghanistan underscore the importance of safe, nurturing environments where engaged teachers, supportive parents, and resourceful communities collectively promote child learning, belonging, and well‐being (Dupree, 2004; Panter‐Brick & Eggerman, 2011; Trani et al., 2019, 2024).

Guided by this evidence, our intervention integrates classroom activities for teachers and students with structured parental engagement to address children's social ecology holistically. Both components emphasize stress management (e.g. breathing exercises, relaxation techniques, conflict resolution), emotional regulation (e.g. recognizing and managing negative emotions), problem‐solving, prosocial behavior, and structured play, which can restore protective environments, reduce stress, and build resilience (Ager et al., 2011; Apfel & Simon, 1996; Betancourt, Meyers‐Ohki, Charrow, & Tol, 2013b). Physical activities – including games, yoga, and mindfulness – aim to reduce stress and build emotional and self‐regulation skills, thereby enhancing psychosocial well‐being and SEL (Diamond & Lee, 2011). The parent component also supports caregivers in managing their own emotional responses and promotes positive, nonpunitive interactions, strengthening their role as a protective buffer for children's mental health (Armstrong, Birnie‐Lefcovitch, & Ungar, 2005; Panter‐Brick, Goodman, Tol, & Eggerman, 2011; Panter‐Brick, Grimon, & Eggerman, 2014). Evidence suggests that when parents struggle to recognize and respond appropriately to children's distress, this can perpetuate children's symptoms (Hafstad, Haavind, & Jensen, 2012).

All materials were translated into Dari and Pashto and culturally adapted. A professional translator reviewed the translations and back‐translated them into English. Intervention content and adaptations were informed by formative research conducted by an Afghan psychologist and several authors and by stakeholder feedback to ensure cultural resonance, acceptability, and feasibility. Adaptations included substituting abstract concepts with concrete examples from children's daily lives; integrating familiar activities such as storytelling, games, and mindfulness practices rooted in local traditions; and revising scenarios. The final package was piloted with teachers, children, and parents in a Kabul school.

We focused on activities for children aged 8–12, using culturally grounded analogies aligned with Afghan values and traditions (Castro, Barrera Jr, & Martinez Jr, 2004). For instance, in Theme 2, ‘I Know About Feelings’, instead of group discussions alone, children pass a ball to one another. The initiating child describes a situation eliciting an emotion (e.g. ‘You are in the dark—how do you feel and why?’), and the recipient reflects and responds (e.g. ‘In the dark I feel afraid because I cannot see’). COVID‐19‐specific activities were removed, and games with random elements were introduced to enhance engagement and spontaneity.

Scenario‐based activities were similarly contextualized. In the original exercise ‘Stranded on a Desert Island’, designed to teach responsible decision‐making, we substituted a locally relevant scenario: a child traveling by car through mountainous or desert terrain experiences a breakdown when two tires are damaged. As night falls, the child must decide whether to stay with the vehicle or seek help and, if leaving, choose one essential item per traveler to bring along.

**Figure 2:**
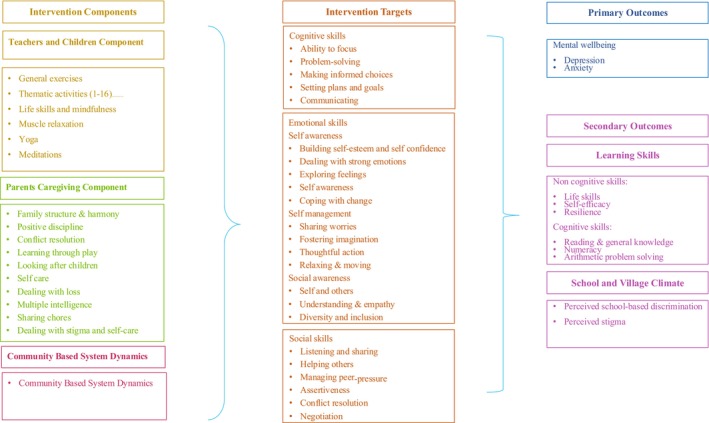
Theory of change of the three‐component social emotional learning intervention

The teacher's guide was developed as a culturally tailored psychosocial intervention targeting anxiety, depressive symptoms, social isolation, and related difficulties among Afghan school children, in a context where such resources remain scarce. Designed to promote cognitive, emotional, and social competencies, the guide equips teachers with structured activities that foster resilience, emotional regulation, self‐awareness, social connectedness, and coping mechanisms for stress and conflict. The intervention comprises 18 thematic modules containing 42 culturally adapted activities that integrate reflective exercises, short lectures, drama, games, mindfulness practices, physical activities, and breathing techniques. Each module begins with introductory exercises, continues with core activities, includes optional energizers to sustain attention, and closes with a decompression activity to release tension and restore emotional balance. Themes include self‐awareness, self‐management, relationship skills, coping with change, decision‐making, and leadership, while also promoting communication, cooperation, analytical thinking, and goal‐setting. Teachers can deliver entire modules as 60–90 min sessions, divide them into weekly segments, or use activities as stand‐alone exercises, adapting implementation to classroom context and time constraints (Figure 2).

The parent engagement component aimed to equip families and caregivers with strategies to enhance family harmony and their capacity to contain children's distress (Jordans, Pigott, & Tol, 2016). The parental toolkit was similarly adapted to local norms governing family life (Dupree, 2004). For instance, in the activity ‘Promoting Pro‐Social Behaviour Rather than Negative Discipline’, we avoided recommendations such as rotating leadership among children to set household rules – an approach inconsistent with norms whereby the primary breadwinner typically enforces family regulations. The parent component consisted of a 1‐day series of activities promoting psychosocial well‐being at home (REPSSI et al., 2021). Training activities addressed time structure (daily home routine, spending family and one‐on‐one time), positive discipline, emotional support and prosocial behaviors, individual self‐care (daily routine, physical activities, connecting with others, accepting feelings, staying hopeful), managing chores, and protecting family harmony. Learning methods emphasized coached practice and small‐group work, using group‐based problem‐solving and mutual support among family members (Figure 2).

We conducted community‐based system dynamics (CBSD) workshops aiming to elicit a shared mental model of childhood happiness and mental well‐being among schools' stakeholders (children, teachers, and parents). CBSD using group model building (GMB) methods enables communities to articulate, prioritize, and connect drivers of distress and resilience using the language of feedback systems (Hovmand, 2014; Rouwette, Vennix, & Mullekom, 2002; Vennix, 1999). Grounded in community‐based participatory research, CBSD elevates community voice and helps navigate power differentials among stakeholder groups (Chambers, 2001). In our application, workshop participants (children, teachers, and parents in different sessions) coconstructed causal maps of child mental well‐being and happiness that integrated environmental, sociocultural, and resource barriers linked to common mental health conditions (e.g. depressive symptoms, anxiety, chronic stress, posttraumatic stress disorder) and adversity (Luthar, Cicchetti, & Becker, 2000). Participants then identified leverage points and proposed actions – explicitly considering components of the ongoing school psychosocial intervention – to reduce disorder burden and strengthen resilience. CBSD was used as a communication mechanism to build buy‐in for our intervention, to justify training school stakeholders in the psychosocial intervention, to assess its cultural resonance and appropriateness, and to give stakeholders a voice in interpreting its components (see Appendix S3 for details).

We adapted structured group exercises from Scriptapedia, an online open‐source collection of GMB scripts tested with school stakeholders (Calhoun et al., 2010). We used the translated idea of child mental well‐being, which was more complicated for workshop participants, particularly children, to grasp. We conducted four distinct activities: (a) focus group discussions to find a shared definition of child mental well‐being; (b) variables elicitation to identify multiple factors influencing child happiness; (c) connections circle to establish connections between factors influencing child happiness, including the identification of causal feedback loops; and (d) action ideas elicitation to intervene on leverage points in the system, including psychosocial activities conducted with teachers and children on one side and parents on the other.

The intervention was delivered by Afghan field‐based educational staff, who were recruited from among the local community and trained on the intervention delivery during a 12‐day‐long training by the authors. After the training, field‐based educational staff delivered several days of mock intervention in a school in Kabul under the supervision of two of the authors. Each team of two field‐based educational staff delivered the intervention in 10 intervention schools, one per province, with two teams in Ghazni, one covering the district of Jaghori specifically. Teachers and parents were actively engaged during the training. The field‐based educational staff oversaw the intervention implemented by teachers in their classrooms and parents at home and provided ongoing support and guidance for the whole duration of the intervention. Both teachers and parents were expected to continue carrying out all activities daily in class and at home. Schools that had been assigned to the control group were on a waitlist to receive the intervention program if found to be effective and if authorities would allow international NGOs to pursue school support.

The intervention was delivered in two intensity conditions to assess dosage effects. Some schools delivered a short‐term intervention (completing all intervention components within 90 days), while others delivered a longer intervention of duration greater than 90 days.

### Procedures

Pre‐intervention data were collected in both intervention and control schools in two waves. In Badakhshan province and Jaghori district of Ghazni province, preintervention data were collected between October 2, 2021 and December 30, 2021, and postintervention data between September 1, 2022 and December 30, 2022. In other districts of Ghazni and in Takhar province, preintervention data were collected between April 10, 2023 and July 23, 2023, and postintervention data between September 3, 2023 and December 25, 2023. After at least 3 months of intervention implementation, where teachers and students practiced activities in schools and parents conducted activities at home, postintervention data were collected in all schools. We recruited and trained six local enumerators with at least a bachelor's degree, led by a supervisor in each of the four study areas. All members of the enumeration team had to pass a final training test. Those with the highest test scores were promoted to a supervisory role. All questionnaires were administered in Dari and Pashto following translation and back‐translation by separate teams of translators.

Due to the nature of the intervention, masking participants and field‐based educational staff who delivered it was not possible. Data collection teams worked independently, at different times and locations, from the field‐based educational staff, and all efforts were made to keep them as masked to group status as possible. Data analysis was blinded. One author checked with enumerators at the end of data collection to elicit how much detail they knew about the intervention and treatment allocation. Results showed they knew little of the program specifics. The fact that all schools had previously been supported by NGO partners facilitated the masking of those who received the psychosocial program from those who received the usual support. For quality assurance, provincial supervisors and the first author randomly supervised 30% of child, teacher, and parent interviews.

### Outcomes

We measured child anxiety and depression using a culturally adapted version of the short version of the Child Generalized Anxiety Disorder Subscale (six items) and the Depression Total Scale (10 items) of the Revised Child Anxiety and Depression Scale short version (Ebesutani et al., 2012). Both measures used a five‐level rating scale ranging from 1 ‘never/not at all’, 2 ‘seldom/a little’, 3 ‘moderately/somewhat/sometimes’, 4 ‘a lot/often/well’ to 5 ‘all the time/almost every day/very well’, and we used saddening faces to help the child choose. Internal reliability (IR) was good with a Cronbach alpha of 0.97 and 0.98 at preintervention and 0.97 and 0.99 at postintervention for anxiety and depression. For the child well‐being domains, we assessed resilience, self‐efficacy, and multiple domains of life skills through adapting several measures: (a) the Child and Youth Resilience Measure (child and youth versions available in Dari; IR = 0.90 and 0.91 at preintervention and postintervention; Ungar & Liebenberg, 2011); (b) the Self‐Efficacy Questionnaire for Children (IR = 0.97 and 0.96 at preintervention and postintervention; Muris, 2001); and (c) the Multidimensional Scale of Life Skills in Late Childhood (IR = 0.96 and 0.97 at preintervention and postintervention; Kobayashi et al., 2013).

Proficiency in reading and mathematics was measured with instruments from the Monitoring Trends in Educational Growth (MTEG) program, developed by the Australian Council for Educational Research (ACER) with the Afghanistan Ministry of Education (Australian Council for Educational Research, 2013). MTEG is a national item response theory‐based framework that tracks grade‐level achievement and longitudinal growth from Grades 3 to 9 by placing items on a developmental difficulty scale.

Mathematics comprised two components: (a) basic numeracy (numeration, operations, introductory algebra) and (b) arithmetic problem‐solving and reasoning via context‐embedded word problems. Reading and general knowledge were assessed through four tasks that targeted letter/word recognition, sentence comprehension, narrative understanding, and interpretation of illustrated vignettes. All tests were multiple‐choice (four options with plausible distractors); scores were treated as continuous counts of correct responses.

Rasch analysis related item difficulty to child ability, assuming higher probabilities of correct responses on easier items (Bond, Yan, & Heene, 2020). Model fit was satisfactory for all three tests (fit statistic −0.99 for reading/general knowledge, basic numeracy, and arithmetic problem‐solving; data not shown).

Stigma, perceived prejudice, and experienced discrimination were assessed using an adapted and validated version of the Discrimination and Stigma Scale developed for India (Brohan et al., 2013) and validated in Afghanistan (IR = 0.73 and 0.6 at preintervention and postintervention). School‐based discrimination was measured using a culturally adapted Discrimination Scale developed for the Maryland Adolescent Development in Context Study (Cogburn, Chavous, & Griffin, 2011).

To reflect the actual socioeconomic status of families, we used both types and numbers of household assets and livestock collected as a proxy of income (Filmer & Pritchett, 2001). We derived an assets and a livestock index from the first factor of two principal components analyses by combining, respectively, the number of different assets (such as radios, generators, and cookers) and the number of livestock (sheep, chickens, horses, etc.) owned by the household (Kolenikov & Angeles, 2009).

### Statistical analysis

Power analysis was based on informed assumptions because no comparable studies have examined the effects of psychosocial interventions among rural Afghan children. Power analyses accounted for the data structure of students nested within schools – the unit of randomization for the intervention – to detect a preintervention standardized effect size (ES) of 0.15 at a one‐tailed alpha of .05 at 80% power, which would require 762 children per group. We used the Abdul Latif Jameel Poverty Action Lab's (JPAL) power calculator to adjust this number for an 80% take‐up in the treatment group, an attrition rate of 10%, 83 clusters (schools), an average cluster size of 20, and an intra‐cluster correlation of .2, which would require a total sample size of 1700 (JPAL Lab, 2024). We recruited a total of 3,827 children, assuring sufficient power for analyses.

Before analyses, to assess randomization success, we checked the balance between intervention and control schools on sociodemographic characteristics (age, gender, ethnicity, school grade, assets index, and livestock index), location, and outcomes at preintervention by applying χ^2^ with continuity correction or Fisher's exact test for frequencies, and independent‐samples *t*‐tests for continuous measures and taking the clustered survey design into consideration (Trani & Hart, under review).

We fitted a three‐level linear model with random intercepts to examine whether students in intervention schools that completed the intervention displayed significantly higher rates of change in anxiety, depression, academic performance, social–emotional outcomes, discrimination, and stigma compared to those in control schools. Student observations pre and postintervention (Level 1) were nested within students (Level 2) who were nested within schools (Level 3). Student‐level variables consisted of baseline characteristics measured prior to treatment exposure (age, sex, ethnicity, assets, and livestock). We included the geographical area (district) of the school as a fixed effect (not shown in the equation). Additionally, we included (a) a school‐level indicator for intervention status, and (b) an interaction term between this intervention indicator and a pre/post dummy variable, allowing us to compare differential rates of change between students in intervention and control schools before and after the intervention.

$$y_{\mathrm{ijt}}=\beta_{0}+\beta_{1}\text{Post}+\beta_{2}\text{Intervention}+\beta_{3}\left(\text{Post}\times \text{Intervention}\right)\;+\beta_{4}^{\prime }X_{\mathrm{ijt}}+\beta_{5}^{\prime }\gamma_{\mathrm{jt}}+u_{0j}+\varepsilon_{\mathrm{ijt}}$$

where

i=1,…,n: child participants.

j=1,…,j schools.

$\text{time}\in \left\{0,1\right\}\;\text{with}\;(0=$ pre‐intervention, 1 = post‐intervention).

$y_{\mathrm{ijt}}$: the outcomes of student *i* in school *j* at time *t*.

Post: time dummy (0 = preintervention, 1 = postintervention).

Intervention: school‐level dummy (1 = intervention school, 0 = control school).

Post×Intervention: interaction capturing the differential pre–post change associated with the intervention.

$X_{\mathrm{ijt}}$: vector of student‐level predictors postintervention, baseline as reference group.

$\gamma_{\mathrm{jt}}$: vector of school‐level predictors.

$u_{0j}$: fixed effect for the school *j*.

$\left(\beta_{0}+u_{0j}\right)$ *:* student random intercept for student *i* within school *j*.

$\varepsilon_{\mathrm{ijt}}$∼*N*(0, *σ*2): Level 1 residual error.

For reasons described in the Discussion section below, schools either delivered shorter‐duration intervention (≤90 days) or longer‐duration intervention (>90 days). This was treated as a school‐level indicator variable (and not shown in the specification above). Preintervention characteristics were compared using χ^2^ tests for categorical variables and independent‐samples *t*‐tests for continuous variables. Intra‐cluster correlations quantified variance attributable to clustering, and *p* < .05 denoted statistical significance.

We estimated the intent‐to‐treat (ITT) effect using a linear hierarchical model with children clustered in schools with fixed effects and random intercepts. We estimated the models using the full sample, and then conducted two sets of subgroup analyses: separately for girls and boys, and separately by geographical area. In order to ensure interpretability across outcomes, we standardized all our outcomes using *z*‐scores [(*X −* mean)/standard deviation (*SD*)] for all our main intervention effects, for both the entire sample and subgroups. Effect sizes were computed for outcomes where significant and positive intervention effects were observed by dividing the unstandardized outcome by the control group baseline *SD*. We used similar approaches for subgroup analyses (data not shown). The multilevel model included preintervention estimates at the individual level. All analyses were done in R software.

## Results

Between September 1, 2022 and December 30, 2022, in Badakhshan and Jaghori districts of Ghazni province and between April 10, 2023 and July 23, 2023 in other districts of Ghazni and in Takhar province, 4,539 eligible participants were identified, of whom 3,827 adolescents aged 8–13 years completed the preintervention survey (1927 assigned to intervention, and 1900 assigned to control), with 81.2% retention at follow‐up (Figure 1). All children in Grades 3–6 in each intervention school were enrolled, and a sample of these were interviewed at preintervention and again at postintervention (the children in Grade 6 were the ones who had been recruited in Grade 5 but had advanced a grade over the summer). The postintervention survey was done at least 3 months after the intervention to leave enough time for the implementation of the activities by teachers and parents. There were significant differences in attrition rates across study groups [201 (16.01%) in the intervention schools, 175 (13.0%) in the control schools; *p* value of joint *F*‐test 0.03].

Intervention and control groups were balanced on participant characteristics and children's preintervention outcomes (Table 1). There were a few more children in Grades 3 and 6 in intervention schools, while more were in Grades 4 and 5 in control schools. There were slightly more children of Tajik and Pashto ethnicity in intervention schools, while close to half of the children in control schools were from a minority background (mostly Hazara and Uzbek). The average livestock index was slightly lower for families in the control group. Reported anxiety and depression symptoms were higher in the control group than in the intervention group at preintervention. The mean scores of *M* = 9.46 (*SD* = 4.29) and *M* = 14.4 (*SD* = 6.56) showed a low average level of anxiety and depression. Conversely, life skills, discrimination, and stigma scores were slightly higher among children in intervention compared to control schools.

**Table 1:** Baseline characteristics of child participants in the intervention and control groups

	Overall (*N* = 3,827)	Control (*N* = 1,900)	Intervention (*N* = 1,927)	*t* Test/*F*	*p* Value
Age (years)	11.5 (1.55)	11.6 (1.55)	11.5 (1.55)	−0.85	.28
Sex					
Male	1,648 (43.1%)	802 (42.2%)	846 (43.9%)	0.06	.80
Female	2,179 (56.9%)	1,098 (57.8%)	1,081 (56.1%)		
Grade					
3	565 (14.8%)	257 (13.5%)	308 (16.0%)	1.15	.32
4	822 (21.5%)	457 (24.1%)	365 (18.9%)		
5	1,205 (31.5%)	632 (33.3%)	573 (29.7%)		
6	1,235 (32.3%)	554 (29.2%)	681 (35.3%)		
Ethnicity					
Tajik	630 (16.5%)	273 (14.4%)	357 (18.5%)	0.43	.64
Pashtun	1,574 (41.1%)	721 (37.9%)	853 (44.3%)		
Other	1,623 (42.4%)	906 (47.7%)	717 (37.2%)		
District					
Ghazni	983 (25.7%)	487 (25.6%)	496 (25.7%)	0.04	.99
Jaghori	639 (16.7%)	326 (17.2%)	313 (16.2%)		
Takhar	1,038 (27.1%)	540 (28.4%)	498 (25.8%)		
Badakhshan	1,167 (30.5%)	547 (28.8%)	620 (32.2%)		
Family Assets Index					
Mean (*SD*)	−0.0262 (1.76)	−0.0602 (1.73)	0.00766 (1.80)	0.26	.79
Animal Index					
Mean (*SD*)	0.123 (1.27)	0.0976 (1.29)	0.148 (1.26)	0.27	.78
Outcomes at baseline					
Anxiety	9.46 (4.29)	9.59 (4.47)	9.33 (4.10)	−0.45	.65
Depression	14.4 (6.56)	14.7 (6.93)	14.1 (6.16)	−0.75	.45
Life skills	52.7 (13.1)	51.9 (13.3)	53.4 (12.8)	−0.65	.53
Resilience	40.3 (5.00)	40.1 (4.96)	40.1 (5.13)	0.76	.44
Self‐efficacy	50.4 (13.5)	50.2 (13.8)	50.6 (13.1)	0.19	.45
Discrimination	0.966 (1.93)	0.780 (1.72)	1.15 (2.10)	0.19	.25
Stigma	3.17 (2.72)	3.06 (2.55)	3.29 (2.89)	0.52	.60
Reading/general knowledge	2.65 (1.33)	2.74 (1.32)	2.57 (1.33)	−1.54	.13
Numeracy	2.51 (0.917)	2.57 (0.912)	2.45 (0.918)	−1.04	.30
Arithmetic problem‐solving	.29 (0.705)	2.31 (0.735)	2.26 (0.727)	−0.67	.50

Data are mean (*SD*) or *n* (%). Significance was adjusted for design effects. *SD*, standard deviation.

Monitoring data on intervention implementation showed that the intervention was delivered as intended, with both 5‐day sessions for teachers and children and 1‐day sessions for parents per intervention school. The proportion of students, teachers, and parents attending workshops was 81.6%, 54.6%, and 60.6%, respectively (Table 2).

**Table 2:** Percentage of children, parents, and teachers directly participating in the psychosocial intervention

	Ghazni	Thakar	Badakhshan	Jaghori	All
Number of students	364	262	344	277	1,247
Received	311 (85.4%)	216 (82.4%)	234 (68.0%)	226 (81.6%)	1,017 (81.6%)
Perceived usefulness	299 (82.1%)	181 (69.1%)	204 (59.3%)	221 (79.8%)	905 (72.6%)
Regular teacher implementation	182 (50.0%)	118 (45.1%)	127 (36.9%)	74 (26.8%)	501 (40.2%)
Number of parents	338	252	281	252	1,123
Received	229 (67.8%)	132 (52.4%)	140 (49.8%)	112 (44.4%)	613 (54.6%)
Perceived usefulness	224 (66.3%)	117 (46.4%)	117 (41.6%)	133 (52.8%)	591 (52.6%)
Number of teachers	30	40	94	62	226
Received	23 (76.7%)	29 (72.5%)	51 (54.3%)	34 (54.8%)	137 (60.6%)
Perceived usefulness	23 (76.7%)	29 (72.5%)	49 (52.1%)	31 (50.0%)	132 (58.4%)
Regular teacher implementation	15 (50%)	21 (50.25%)	38 (40.4%)	10 (16.2%)	84 (37.2%)

Attrition analyses did not reveal significant differences between retained and lost cases on demographic characteristics collected preintervention (Table S1). There were significant differences in preintervention outcomes. Life skills, self‐efficacy, numeracy, and arithmetic problem‐solving were significantly lower among those who left school, while anxiety, depression, and stigma were significantly higher. These results informed our ITT analyses, below. In the Discussion section, we explain these results in the context of the Taliban takeover of Afghanistan and the resultant limitations for our study.

### Overall treatment effect

Children in the intervention group showed no significant differences in terms of anxiety and depression reduction scores at postintervention compared to children in the control group (Table 3). Intervention schools demonstrated significantly higher life skills scores than control schools at both pre‐ and postintervention. However, due to their higher baseline levels, the magnitude of improvement was smaller – intervention schools improved 0.11 *SD* less than control schools (*p* < .001).

**Table 3:** Effects of intervention on child mental well‐being outcomes, school‐based stigma and discrimination, depression, anxiety, and academic outcomes, including covariates

Outcomes	Intervention			Wave			Intervention × wave		
	Estimates	CI	*p* Value	Estimates	CI	*p* Value	Estimates	CI	*p* Value
Life skills	0.25	0.14 to 0.36	**<.001**	0.06	0.005 to 0.11	.**032**	−0.11	−0.18 to −0.03	.**004**
Resilience	0.07	−0.04 to 0.19	.183	0.07	0.02 to 0.12	.**007**	−0.03	−0.11 to 0.04	.342
Self‐efficacy	0.08	−0.04 to 0.20	.179	0.01	−0.05 to 0.06	.802	−0.01	−0.09 to 0.07	.765
Stigma	−0.001	−0.13 to 0.13	.994	−0.19	−0.25 to −0.13	**<.001**	0.04	−0.05 to 0.12	.391
Discrimination	0.35	0.21 to 0.49	**<.001**	−0.20	−0.26 to −0.13	**<.001**	−0.16	−0.25 to −0.07	.**001**
Depression	−0.08	−0.22 to 0.05	.218	−0.26	−0.32 to −0.20	**<.001**	0.002	−0.08 to 0.09	.968
Anxiety	−0.06	−0.19 to 0.08	.401	−0.23	−0.29 to −0.17	**<.001**	0.0005	−0.09 to 0.09	.990
Reading/general knowledge	−0.33	−0.43 to −0.23	**<.001**	0.18	0.14 to 0.23	**<.001**	0.22	0.16 to 0.29	**<.001**
Numeracy	−0.30	−0.41 to −0.20	**<.001**	0.19	0.14 to 0.23	**<.001**	0.21	0.15 to 0.27	**<.001**
Arithmetic problem‐solving	−0.14	−0.25 to −0.02	.**023**	0.09	0.03 to 0.14	.**001**	0.09	0.02 to 0.17	.**014**

Standard errors are clustered at the school level. Age, gender, ethnicity, district, assets, and livestock were used as control variables. CI, confidence interval. Significant p value for coefficient at *p* < 0.05 or smaller are highlighted.

**Figure 3:**
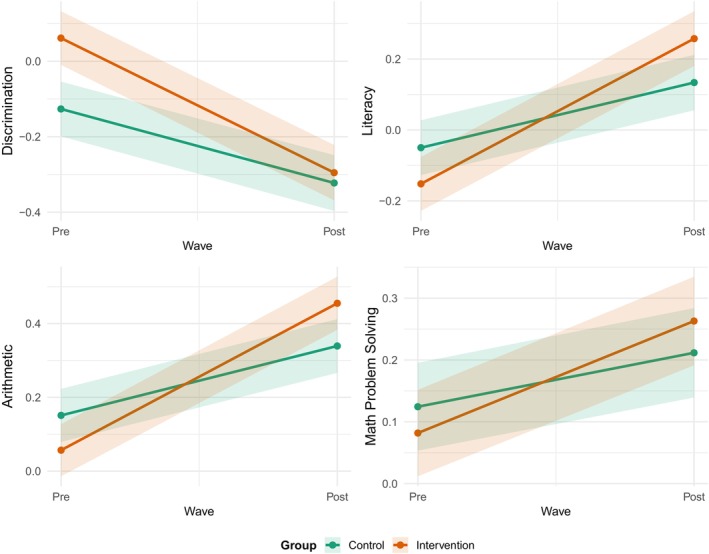
Outcomes showing significant differential change other time between intervention and control schools

We also found a significant intervention effect on reading/general knowledge (0.22 *SD*, *p <* .001), numeracy (0.21 *SD*, *p <* .001), and arithmetic problem‐solving (0.09 *SD*, *p* = .014). School‐based discrimination diminished significantly (−0.16 *SD*; *p* = .001) but stigma did not, comparing children in intervention and control schools (see Figure 3).

### ESs of significant changes in outcomes of the overall treatment effect

Table 4 shows the ESs for all outcomes with a positive impact. First, students in intervention schools showed a greater reduction from pre‐ to postintervention in school‐based discrimination: 0.27 more points compared to controls (β = −.27, *SE* = 0.08). This corresponds to a small difference‐in‐differences (DID) ES of −0.16. Although the intervention group had a higher mean discrimination score than controls postintervention, they demonstrated a larger reduction across the intervention period. Second, the Intervention × Wave estimate was positive (β = .30, *SE* = 0.04), yielding a standardized DID ES of 0.23 for reading/general knowledge. We also found a significant improvement for intervention schools (β = .20, *SE* = 0.03, ES = 0.22) for numeracy. Finally, arithmetic problem‐solving showed a modest but positive Intervention × Wave effect (β = .07, *SE* = 0.03, ES = 0.09).

**Table 4:** Effect sizes of intervention on child mental well‐being outcomes, school‐based stigma and discrimination, depression, anxiety, and academic outcomes, including covariates

Outcomes	Intervention × wave coefficient (*SE*)	Control group *SD*	Differences‐in‐differences effect size
School‐based discrimination	−0.27 (0.08)	1.72	−0.16
Reading/general knowledge	0.30 (0.04)	1.32	0.23
Numeracy	0.20 (0.03)	0.91	0.22
Arithmetic problem‐solving	0.07 (0.03)	0.74	0.09

Effect sizes are standardized on the control group standard deviation. Age, gender, ethnicity, district, assets, and livestock were used as control variables. *SD*, standard deviation; *SE*, standard error.

### Intervention duration effects

Intervention duration was associated with differential psychosocial improvements over time (Table S2). Findings suggested that children at intervention schools that delivered shorter interventions (<90 days) displayed significantly more reductions in depression (−0.18 *SD*, *p* = .003) and anxiety (−0.24 *SD*, *p* < .001), and a significant increase in life skills (0.11 *SD*, *p* = .036) compared to children in control schools, suggesting early benefits in adaptive functioning. There was no significant effect on resilience. Children in schools delivering longer‐duration interventions showed a significant increase in anxiety (0.17 *SD*, *p =* .001) and a borderline significant increase in depression compared to children in control schools, although both of small magnitude. We also found less improvement in life skills (−0.30 *SD*, *p* < .001) and self‐efficacy (−0.13, *p* < .003) compared to control schools.

**Figure 4:**
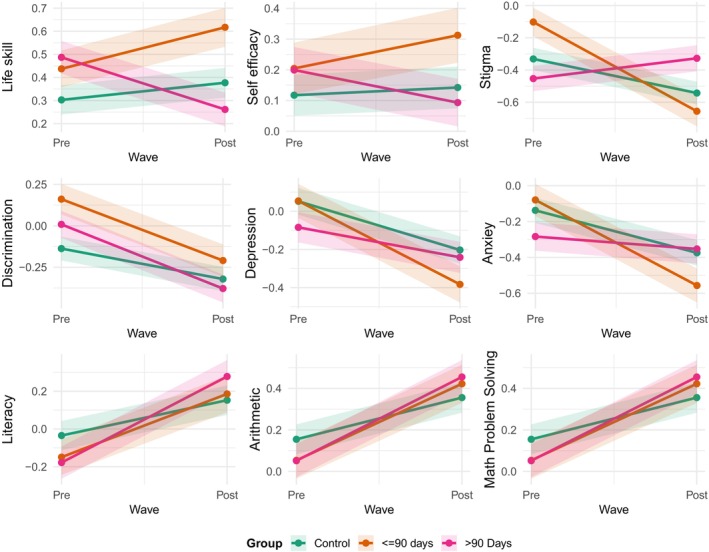
Differential change in all outcomes over time between intervention and control schools according to the duration of the intervention

Reading/general knowledge and numeracy improved more in schools with both shorter (respectively, *SD* 0.15, *p* = .001 and 0.17 *SD*, *p* < .001) and longer interventions (respectively, *SD* 0.27, *p* < .001 and 0.20 *SD*, *p* < .001) compared to control schools, indicating a persistent and increasing effect. Arithmetic problem‐solving improved significantly more in short‐term intervention schools only (0.14 *SD*, *p* = .01; see Figure 4).

Stigma and discrimination decreased more in schools with shorter interventions (respectively, −0.34 *SD*, *p* < .001 and −0.19 *SD*, *p* = .003), but only discrimination continued to decrease more with a longer intervention (−0.20, *p* < .001). Stigma increased in schools with longer interventions compared to control schools (0.34 *SD*, *p* < .001).

### Intervention effects by gender

Girls in intervention schools exhibited significantly less reduction in depression (0.13 *SD*, *p* = .027), anxiety (0.14 *SD*, *p* = .017), and stigma (0.17 *SD*, *p* = .005), and less improvement in life skills (−0.23 *SD*, *p* < .001) than in control schools. Conversely, academic outcomes improved significantly more: reading/general knowledge by 0.22 *SD* (*p* < .001) and numeracy by 0.11 *SD* (*p* = .014). Self‐efficacy, resilience, discrimination, and arithmetic problem‐solving did not differ significantly (Table S3). In dose analyses, girls in schools receiving both shorter and longer interventions improved more in reading/general knowledge compared to girls in control schools (Table S4). Discrimination was reduced further for girls receiving longer interventions compared to girls in control schools (marginally significant for girls in schools with shorter interventions). However, anxiety, depression, and stigma decreased significantly less while life skills and self‐efficacy improved significantly less among girls in schools with longer interventions compared to control schools.

Results were more encouraging for boys. Symptoms of depression (−0.18 *SD*, *p* = .007) and anxiety (−0.19 *SD*, *p* = .005), as well as perceived discrimination (−0.21 *SD*, *p* = .002), significantly declined, while reading/general knowledge (0.25 *SD*, *p* < .001), numeracy (0.36 *SD*, *p* < .001), and arithmetic problem‐solving (0.26 *SD*, *p* < .001) increased. Yet, no statistically significant differences in improvement were observed in life skills, self‐efficacy, resilience, and stigma compared to boys in control schools (Table S5). In schools receiving the shorter intervention, anxiety and depression, as well as stigma and discrimination, decreased substantially more than in control schools, while life skills, self‐efficacy, reading/general knowledge, numeracy, and arithmetic problem‐solving improved more. Academic outcomes also improved more in schools with longer interventions, and discrimination kept decreasing in those schools compared to control schools. However, life skills improved less in schools with longer interventions compared to control schools, while stigma decreased less, indicating, respectively, a possible ceiling and floor effect for those two outcomes (Table S6).

### Intervention effects by area

In Badakhshan province, students in intervention schools demonstrated greater reduction in anxiety (−0.26 *SD*, *p* = .006) but not in depression compared to students in control schools. Significantly greater improvements were observed in life skills (0.65 *SD*, *p* < .001) and self‐efficacy (0.70 *SD*, *p* < .001), but not in resilience. For academic outcomes, significantly larger improvements were observed in reading/general knowledge (0.39 *SD*, *p* < .001), numeracy (0.49 *SD*, *p* < .001), and arithmetic problem‐solving (0.42 *SD*, *p* < .001). Significantly greater reductions were observed in stigma (−0.46 *SD*, *p* < .001) and discrimination (−0.19 *SD*, *p* = .045; Table S7a). Anxiety and depression both significantly decreased more in short‐term interventions compared to control schools. Furthermore, short‐ and long‐term interventions were associated with sustained psychosocial and academic improvements as well as a reduction in discrimination. Finally, stigma decreased significantly more in schools with shorter interventions compared to control schools, but not in schools with longer interventions (Table S7b).

In Ghazni province, students in intervention schools demonstrated significantly less reduction in anxiety (0.26 *SD*, *p* = .002) and depression symptoms (0.26 *SD*, *p* = .002), as well as smaller improvements in life skills (−0.38, *p* < .001) and self‐efficacy (−0.27 *SD*, *p* < .001) compared to students in control schools. Similarly, fewer gains were observed for arithmetic problem‐solving (−0.24 *SD*, *p* < .001), but there were no statistically significant differences in gains across reading/general knowledge and numeracy (Table S8a). Schools with longer interventions saw anxiety and depression reducing less than in control schools. Schools with both shorter and longer interventions showed less improvement in life skills and self‐efficacy than control schools. Academic outcomes did not improve more with the longer duration of the intervention. Finally, stigma was reduced less in intervention schools than in control schools for both intervention lengths. Only discrimination decreased significantly more in longer‐intervention schools (Table S8b).

In Jaghori district of Ghazni province, the intervention showed no significant effect on anxiety and depression. Students in intervention schools made smaller improvements in life skills (−0.27 *SD*, *p* = .017) and self‐efficacy (−0.24 *SD*, *p* = .030) compared to students in control schools. Intervention schools did not demonstrate significantly greater improvements in any academic outcome compared to control schools. Finally, there was significantly more reduction in stigma (−0.33 *SD*, *p* = .003) but smaller reduction in discrimination (0.31 *SD*, *p* = .007) compared to control schools (Table S9a). The length of the intervention did not influence the effect on anxiety and depression. Self‐efficacy improved significantly less in shorter‐intervention schools, and life skills in longer‐intervention schools, compared to control schools. There was no significant difference in effect between schools, whatever the duration of the intervention, on resilience and academic outcomes. Stigma decreased more in shorter‐intervention schools than in controls, but discrimination did not for both intervention lengths (Table S9b).

In Takhar province, intervention schools demonstrated significantly greater reduction in depression (−0.28 *SD*, *p* = .001) but not in anxiety, as well as less improvement in life skills (−0.30 *SD*, *p* < .001) and self‐efficacy (−0.16 *SD*, *p* = .040) compared to control schools, indicating less improvement in these psychosocial outcomes over time. However, they showed greater improvement in all academic outcomes: reading/general knowledge (0.45 *SD*, *p* < .001), numeracy (0.41 *SD*, *p* < .001), and arithmetic problem‐solving (0.23 *SD*, *p* = .004). Intervention schools showed less reduction in stigma (0.49 *SD*, *p* < .001) but greater reduction in discrimination (−0.35 *SD*, *p* < .001; Table S10a) compared to controls. Longer interventions resulted in significantly lower depression but not anxiety compared to controls. Children in shorter‐intervention schools demonstrated significantly greater increases in self‐efficacy. All academic outcomes also significantly improved more in intervention schools than in control schools, whatever the length of the intervention. Discrimination was reduced more in longer‐intervention schools compared to controls, but not stigma (Table S10b).

## Discussion

In this cluster‐randomized controlled trial, we evaluated a classroom‐based, participatory psychosocial intervention designed to reduce symptoms of common mental disorders, strengthen well‐being, social–emotional and academic learning skills, and reduce perceived school‐based discrimination and community stigma among children in Afghanistan. The trial unfolded during a period of profound political upheaval. As the intervention was being implemented, the Republic of Afghanistan collapsed following the withdrawal of US‐led forces, and the Taliban established the Islamic Emirate. The trial did not demonstrate consistent improvements in anxiety, depression, or other psychosocial outcomes once we accounted for geographical heterogeneity and gender, whereas it did yield robust gains in reading/general knowledge, numeracy, and arithmetic problem‐solving across three provinces. Overall, in the context of regime change, children's mental health may have been more directly shaped by escalating threats to safety, restrictions on girls' education, and uncertainty about the future – factors that can offset or even reverse psychosocial benefits, particularly for girls.

These contextual shifts also generated unplanned variation in the duration and intensity of intervention delivery. In the first phase of the study, in Badakhshan and Jaghori, headmasters and teachers were often able to ignore the directive to exclude girls beyond Grade 6 and, with tacit approval from district and provincial authorities, implement the full set of activities within a relatively compressed time frame.

In the second phase, in Ghazni and Takhar, the Ministry for the Propagation of Virtue and the Prevention of Vice exerted increasing control over schools, especially those serving girls. Teachers and headmasters faced mounting pressure to comply with restrictions on girls' education and curriculum changes, rendering implementation more fragmented, stressful for children, and slower to complete. As a result, nine of 10 schools in Takhar and six of nine in Ghazni required longer calendar time to deliver the planned content.

In Takhar, implementation remained precarious because authorization was secured locally but not provincially. In Ishkamish and Worsaj districts, district education authorities regularly stopped our activities under multiple pretexts (asking for multiple meetings with authorities, asking to see the program, etc.). Another complication that occurred in Takhar was that the burning of the Holy Quran in Sweden in July 2023 resulted in the Swedish Committee for Afghanistan, our original field partner in that province, being asked to terminate its activities. Consequently, our other field partner, the Norwegian Afghanistan Committee, took over intervention delivery in the schools, resulting in a delay.

In the three remaining girls' schools in Ghazni's conservative Qarabagh and Moqur districts, local authorities' reluctance to authorize our female trainers substantially curtailed the duration of the intervention. Consequently, our results play against on a unique backdrop of regime change, and several of our findings and limitations can be directly attributed to the context within which we, our collaborators and field partners, and our study participants all found ourselves.

We found that our intervention significantly reduced child anxiety and depression in schools implementing the intervention for up to 90 days, but not in schools with longer interventions (>90 days). A recent systematic review of school‐based programs for child anxiety and depression in LMICs found that relatively few interventions, predominantly cognitive‐behavioral, achieved significant benefits, typically over brief periods (Dieu Yin et al., 2025). The apparent ‘dose–response’ pattern for mental disorders reflects the changing political context rather than a simple effect of intervention length. Under comparatively stable conditions, teachers and parents at home could deliver the full set of activities with reasonable continuity, and we observed significant reductions in anxiety and depression. We did not find a significant effect on resilience in schools with either shorter or longer interventions. Compared to students in control schools, students in schools with shorter interventions showed greater improvement in life skills, while those in schools with longer interventions showed less improvement in both life skills and self‐efficacy. School‐based discrimination, but not family and community stigma, which was minimal at preintervention, was significantly decreased by our intervention. Both significantly decreased in schools with shorter interventions. Our intervention improved reading/general knowledge and numeracy in all intervention schools, regardless of the length of the intervention, as well as arithmetic problem‐solving in schools with shorter interventions. The core components of the intervention that targeted classroom practices, cooperative learning, and problem‐solving may have continued to support academic performance despite ongoing and worsening stressors.

In our trial, benefits were larger for boys, inconsistent with some evidence that school‐based SEL can yield greater gains for girls (Ager et al., 2011; Tol et al., 2008). One plausible mechanism is that classrooms offered boys a comparatively safer space to express distress, whereas girls faced more constrained opportunities – particularly the Taliban‐led Emirate's restrictions on girls' education that were instituted and then more strictly implemented progressively in all schools during the study period, as an application of the *Purdah*, sex‐segregation rules that restrict girls' and women's access to public spaces (Dupree, 1992). More broadly, substantial social and environmental upheaval – disproportionately affecting girls and ethnic minorities – likely attenuated overall mental health effects. Exposure to these effects was heightened as our study progressed, and this interaction between an intervention designed to improve outcomes among children and an environment designed to mitigate it for large segments of our participants perhaps accounts for some of the reasons why a longer‐duration intervention was not always effective.

Our findings contrast with another study conducted in conflict‐affected Nepal that combined creative‐expressive and experiential therapy, cooperative play, and cognitive behavioral therapy but detected no significant effects on anxiety and depression after accounting for school‐level clustering (Jordans et al., 2010). Differences in crisis intensity, baseline vulnerability, and implementation stability likely contribute to this divergence, as these factors jointly shape the effectiveness of school‐based psychosocial interventions. In our setting, CBSD ceded ownership to teachers, students, and parents, enabling them to judge the relevance of training components and calibrate intensity accordingly, which may have been critical in generating the gains we observed.

We observed no overall effects on child well‐being outcomes (resilience, self‐efficacy, and life skills). Only life skills improved more in schools receiving the shorter intervention compared to control schools. By contrast, academic outcomes improved overall. These patterns likely reflect the conjunction of conditions required for sustained gains in social–emotional and academic learning – conditions that were difficult to achieve amid conflict and regime change. First, effective regulation of behavior and emotion is a prerequisite for progress in both social–emotional (Durlak et al., 2011) and academic domains (Kim, Brown, Tubbs Dolan, Sheridan, & Aber, 2020). Second, learning is fostered by a supportive ecosystem in which adults (teachers, parents) and peers engage consistently and constructively, including in conflict settings (Trani et al., 2024, 2025), and by parental investments in childhood education (Cunha & Heckman, 2008). Third, academic gains depend on conducive classroom conditions – manageable class sizes, equitable resources, high‐quality instruction, caring teacher practices, and positive teacher–student relationships (Cantor, Osher, Berg, Steyer, & Rose, 2019; Darling‐Hammond, Flook, Cook‐Harvey, Barron, & Osher, 2020; Osher, Cantor, Berg, Steyer, & Rose, 2020; Podolsky, Kini, Darling‐Hammond, & Bishop, 2019; Zaman, Khurram, Alwi, & Shaiq, 2019). Finally, progress requires a tolerant environment, free from discrimination and stigma, which in Afghanistan disproportionately affects girls and children with disabilities (Kissane, 2012; Trani et al., 2019).

Social–ecological outcomes (school‐based discrimination and community stigma) significantly improved with a small effect, and mostly for boys. Discriminatory behaviors are widespread community practices, including physical punishment in class and domestic violence against women and children (Catani, Schauer, & Neuner, 2008; Li, Rao, Natiq, Pasha, & Blum, 2018). Furthermore, stigma and discrimination increase dropout, and this is why children lost to follow‐up had higher scores at baseline. Finally, reducing stigma and discrimination entails long‐term strategies of sensitization and interpersonal contact aimed at shifting mindsets, and these conditions were not met in the study, even in schools with longer‐term interventions (Corrigan, Michaels, & Morris, 2015).

Marked provincial heterogeneity emerged. The intervention produced its strongest and most consistent benefits in academic domains (reading/general knowledge, numeracy, and arithmetic problem‐solving), with substantial gains across three of the four areas. Psychosocial and social–ecological outcomes (anxiety, depression, life skills, self‐efficacy, stigma, and discrimination) showed greater heterogeneity, with the largest gains in Badakhshan and more modest or negative effects in Ghazni, Jaghori, and Takhar. Children in Badakhshan had more significant gains compared to peers elsewhere on most outcomes, likely because this remote province was comparatively insulated from Taliban interference, and the intervention was implemented in the first phase, before intensified oversight by the Ministry for the Propagation of Virtue and the Prevention of Vice. In Jaghori, despite early implementation, children in intervention schools did not show greater improvements in mental well‐being, life skills, self‐efficacy, resilience, discrimination, or academic outcomes compared to children in control schools, plausibly reflecting heightened distress among the predominantly Hazara population, a minority historically persecuted by the Taliban (Amin & Muhammad, 2023; Saleem & Tarar, 2024). However, they did show greater reductions in community stigma, but mostly in schools that implemented the shorter intervention, which may indicate that the intervention successfully reduced stigma in the short term, but the progress was undone or even reversed by the escalated persecution and instability following the Taliban takeover. In Takhar, children in intervention schools showed greater improvements in depression, discrimination, and academic performance, consistent with lower baseline levels – signaling greater initial need and vulnerability – and strong stakeholder engagement sustaining implementation over a longer period, despite authorities' ongoing restrictions, particularly in Ishkamish and Worsaj districts. By contrast, children in intervention schools in Ghazni did not show greater gains compared to children in control schools, except for reduced discrimination in longer‐term intervention schools. Ghazni posed special challenges for program implementation. Increasing restrictions on research in that province, and especially in the conservative districts of Qarabagh and Moqur, resulted in restrictions on the work of our women trainers and increasing scrutiny of all intervention‐related activities. Pressures upon students, teachers, parents, and NGO staff that resulted from such external surveillance may have worsened outcomes among children in that province despite our best efforts. Overall, these patterns suggest that while school‐based psychosocial interventions can retain their effectiveness during major crises, their impact depends critically on the degree to which schools can maintain stable delivery under intensifying restrictions.

To our knowledge, our study is the largest in a conflict setting to evaluate the effectiveness of an intervention to improve child mental health delivered by nonspecialized, field‐based educational staff. This task‐shifting approach was a specific feature of the intervention design, given the paucity of trained mental health professionals in Afghanistan in general and in rural Afghanistan in particular. A local NGO managed the intervention with nonspecialist interventionists recruited from the community and trained by authors using minimal resources following a suggested strategy for closing the treatment gap (Patel et al., 2018). Many of these field staff were young graduates, and it was their first professional experience. Our findings suggest that community‐recruited, field‐based educational staff are fully capable of delivering interventions such as ours, with significant effects on child mental well‐being. These results enhance the possibility of successful implementation (replication and scaling up) in other regions within Afghanistan or other conflict‐affected contexts.

Despite these strengths, our study has some limitations. First, we measured the combined effect of both the classroom‐based and the parent‐focused intervention components. In this study, we did not independently test the efficacy of these two components using a dismantling design. Our decision was based on the fact that existing studies in conflict settings have shown the limited effect of single‐component interventions, while combined interventions engaging teachers, children, and parents have shown an impact (Ager et al., 2011). We propose to test this decision in future work using a factorial design, such as multiphase optimization approaches. Second, we used self‐reported measures of child mental health, resilience, self‐efficacy, life skills, discrimination, and stigma. The reason is feasibility–working in rural Afghanistan, we simply did not have access to trained mental health professionals who could serve as clinical evaluators or diagnosticians. While these measures are widely used in conflict and crisis contexts, we recognize that they may be subject to self‐report bias. Third, in this study, we did not test the mechanisms of action of our intervention (such as trauma processing and exposure or behavioral activation) that could explain intervention effects. To do that, we would have needed different training for the NGO staff involved, and we lacked the resources to test such mechanisms on a similar large scale as our school‐based intervention. Fourth, we analyzed composite indices of anxiety, depression, life skills, self‐efficacy, and resilience rather than their subcomponents. Future trials should be powered and prespecified to examine key subdomains, to clarify which dimensions of psychosocial functioning are most sensitive to political upheaval and gendered constraints on schooling. Fifth, monitoring the intervention to document fidelity was challenging, given the state of the Afghan education system. Trainers conducted follow‐up visits to check compliance with the training provided, but fidelity assessments had to be curtailed because of the change in regime and deterioration in the security environment for our in‐house team and Western researchers. We interviewed participants at postintervention about the frequency of teachers' and parents' engagement in the different activities and about their usefulness. We found high consensus about the intervention's positive effect on child mental well‐being (Trani and Hart, under review). However, we are unable to conduct a formal fidelity analysis due to the unique challenges of conducting research in Taliban‐administered Afghanistan. Finally, we experienced attrition, and participants who were lost to follow‐up were worse off than those who remained. In our study, we are unable to fully model the effects of attrition upon intervention effects, and present ITT estimates as a conservative way to quantify treatment effects.

Despite these challenges, our findings suggest that school‐based psychosocial interventions can be feasibly deployed in conflict and crisis contexts with promising results on child mental health. As such, they are a testimony to the resilience of Afghan children who continue to face significant challenges to their learning (and, indeed, survival) in contemporary Afghanistan. Expansions of these kinds of strategies to other regions of Afghanistan offer a reasonable approach to ensure that Afghan children are protected to the extent possible from the existential challenges that they continue to face every day. This approach is also relevant for other ongoing conflict and crisis contexts.

## Ethical considerations

All participants provided written (or witnessed, if they were unable to write) informed consent. The study was approved by the Norwegian Afghanistan Committee Board after consultation with the Ministry of Education and the Ministry of Higher Education of Afghanistan on September 17, 2022, and by the Human Research Protection Office at Washington University in St. Louis on September 9, 2022 (#201712020).

## Trial registration

This trial has been registered on November 27, 2024 with the International Standard Randomized Controlled Trials Number registry (ISRCTN83632872; https://www.isrctn.com/trialist).

**Key points**

**What is known?**

- Current evidence of school or classroom‐based psychosocial interventions to improve child mental health and learning outcomes in conflict and crisis contexts is limited and lacks a strong scientific basis.

**What is new?**

- We found that a classroom‐based intervention providing culturally adapted psychosocial support engaging teachers, parents, and children and provided by lay community workers improves academic outcomes and reduces discrimination. Shorter interventions reduce child anxiety and depression in conflict and crisis.

**What is relevant?**

- Further research is required to determine whether classroom‐based interventions can effectively provide psychosocial support to a broad population of children in emergency settings and mitigate the adverse mental health impacts of ongoing economic, political, and social crises.

## Supporting information


**Appendix S1.** Content of the intervention for teachers and children.
**Appendix S2.** Content of the intervention for parents.
**Appendix S3.** Training procedure and schedule.
**Table S1.** Comparison between children who stayed and dropped out.
**Table S2.** Effects of intervention length on child mental well‐being outcomes, school‐based stigma and discrimination, depression, anxiety, and academic outcomes, including covariates.
**Table S3.** Effects of intervention on girls' mental well‐being outcomes, school‐based stigma and discrimination, depression, anxiety, and academic outcomes, including covariates.
**Table S4.** Effects of intervention length on girls' mental well‐being outcomes, school‐based stigma and discrimination, depression, anxiety, and academic outcomes, including covariates.
**Table S5.** Effects of intervention on boys' mental well‐being outcomes, school‐based stigma and discrimination, depression, anxiety, and academic outcomes, including covariates.
**Table S6.** Effects of intervention length on boys' mental well‐being outcomes, school‐based stigma and discrimination, depression, anxiety, and academic outcomes, including covariates.
**Table S7a.** Effects of intervention on child mental well‐being outcomes, school‐based stigma and discrimination, depression, anxiety, and academic outcomes, including covariates in Badakhshan.
**Table S7b.** Effects of intervention length on child mental well‐being outcomes, school‐based stigma and discrimination, depression, anxiety, and academic outcomes, including covariates in Badakhshan.
**Table S8a.** Effects of intervention on child mental well‐being outcomes, school‐based stigma and discrimination, depression, anxiety, and academic outcomes, including covariates in Ghazni.
**Table S8b.** Effects of intervention length on child mental well‐being outcomes, school‐based stigma and discrimination, depression, anxiety, and academic outcomes, including covariates in Ghazni.
**Table S9a.** Effects of intervention on child mental well‐being outcomes, school‐based stigma and discrimination, depression, anxiety, and academic outcomes including covariates in Jaghori.
**Table S9b.** Effects of intervention length on child mental well‐being outcomes, school‐based stigma and discrimination, depression, anxiety, and academic outcomes, including covariates in Jaghori.
**Table S10a.** Effects of intervention on child mental well‐being outcomes, school‐based stigma and discrimination, depression, anxiety, and academic outcomes, including covariates in Takhar.
**Table S10b.** Effects of intervention length on child mental well‐being outcomes, school‐based stigma and discrimination, depression, anxiety, and academic outcomes, including covariates in Takhar.

## Data Availability

The data that support the findings of this study are openly available in UK Data Service ReShare at http://www.ukdataservice.ac.uk/citethedata.
