# Supplementary material for: Improving child mental health and learning outcomes and reducing stigma and discrimination in conflict setting: findings from a cluster randomized controlled trial of a classroom‐based psychosocial intervention in rural primary schools in Afghanistan
Source: J Child Psychol Psychiatry. 2026 Jan 25;67(7):1127–46. doi: 10.1111/jcpp.70125 (PMC13265634; doi:10.1111/jcpp.70125)
Supplement: Supplementary file 1 — Appendix S1. Content of the intervention for teachers and children. Appendix S2. Content of the intervention for parents. Appendix S3. Training procedure and schedule. Table S1. Comparison between children who stayed and dropped out. Table S2. Effects of intervention length on child mental well‐being outcomes, school‐based stigma and discrimination, depression, anxiety, and academic outcomes, including covariates. Table S3. Effects of intervention on girls' mental well‐being outcomes, school‐based stigma and discrimination, depression, anxiety, and academic outcomes, including covariates. Table S4. Effects of intervention length on girls' mental well‐being outcomes, school‐based stigma and discrimination, depression, anxiety, and academic outcomes, including covariates. Table S5. Effects of intervention on boys' mental well‐being outcomes, school‐based stigma and discrimination, depression, anxiety, and academic outcomes, including covariates. Table S6. Effects of intervention length on boys' mental well‐being outcomes, school‐based stigma and discrimination, depression, anxiety, and academic outcomes, including covariates. Table S7a. Effects of intervention on child mental well‐being outcomes, school‐based stigma and discrimination, depression, anxiety, and academic outcomes, including covariates in Badakhshan. Table S7b. Effects of intervention length on child mental well‐being outcomes, school‐based stigma and discrimination, depression, anxiety, and academic outcomes, including covariates in Badakhshan. Table S8a. Effects of intervention on child mental well‐being outcomes, school‐based stigma and discrimination, depression, anxiety, and academic outcomes, including covariates in Ghazni. Table S8b. Effects of intervention length on child mental well‐being outcomes, school‐based stigma and discrimination, depression, anxiety, and academic outcomes, including covariates in Ghazni. Table S9a. Effects of intervention on child mental well‐being outcomes, school‐based [file JCPP-67-1127-s001.docx]

**Improving child mental health and learning outcomes and reducing stigma and discrimination in a conflict setting: Findings from a cluster randomized controlled trial of a classroom-based psychosocial intervention in rural primary schools of Afghanistan**

**SUPPORTING INFORMATION**

1. **Appendix S1. Training procedure and schedule**

| ***Sessions*** | Activities | Trainers |
| --- | --- | --- |
|  | ***Day 1 : Sunday May 15th*** |  |
| *0800-0815* | *Opening the training workshop/blessing* | *TBD* |
| *0815-0830* | *Introduction of participants* | *All* |
| *0830-0840* | *Agree with the class on a sign to instruct the class to keep quiet.* | *All* |
| *0840-0900* | *Overview of project* | *JFT* |
| *0900-0915* | *Mindfulness: Progressive Muscle Relaxation for Children* | *IK/JFT* |
| *0915-1015* | *Theories Child mental health, resilience* | *Sahar* |
| *1015-1030* | *Tea break* |  |
| *1030-1130* | *Theories Child mental health, resilience* | *Sahar* |
| *1130-1200* | *Self awareness* | *JFT/IK* |
| *1230-1330* | *Lunch* |  |
| *1330-1345* | *Slow motion fast forward* | *All* |
| *1345-1415* | *Self management* | *IK/JFT* |
| *1415-1445* | *Social awareness* | *JFT/IK* |
| *1445-1500* | *Tea break* |  |
| *1500-1530* | *Relationship skills* | *IK/JFT* |
| *1530-1615* | *Responsible decision making* | *JFT/IK* |
| *1615-1630* | *Evolution (Mindfullness)* | *All* |
|  | ***Day 2: Monday May 16th*** |  |
| *0815-0830* | *If It’s True for You (Mindfullness)* | *JFT/IK* |
| *O830-0845* | *Feedback on yesterday session* | *All* |
| *0845-0930* | *Activities not finished day 1* | *IK/JFT* |
| *0930-1000* | *Circle time:* |  |
| *1000-1015* | *Tea break* |  |
| *1015-1100* | *Yes I made a mistake* | *Sahar/JFT/IK* |
| *1100-1130* | *Theme 1. My school today: Appreciation* | *IK/JFT/Sahar* |
| *1130-1200* | *Theme 2 I know about feelings: Feelings and why we have them* | *JFT/Sahar/IK* |
| *1200-1230* | *Theme 2 I know about feelings: Identifying important (core) feelings* | *Sahar/IK/JFT* |
| *1230-1330* | *Lunch* |  |
| *1330-1345* | *Like a tree* | *JFT* |
| *1345-1415* | *Theme 2: I know about feelings: Wall of feelings* | *JFT/Sahar/IK* |
| *1415-1445* | *Theme 2 I know about feelings: X and y gets angry, sad, nervous, and afraid: Reasons for feeling* | *IK/JFT/Sahar* |
| *1445-1500* | *Tea Break* |  |
| *1500-1530* | *Theme 2 I know about feelings: Changing my mood* | *Sahar/IK/JFT* |
| *1530-1600* | *Theme 3 I help others. Today, I will help…* | *JFT/Sahar/IK* |
| *1615-1630* | *The gift from anger* | *IK/JFT/Sahar* |
|  | ***Day 3: Tuesday May 17th*** |  |
| *0815-0830* | *Magic Carpet* | *IK/JFT* |
| *0830-0845* | *Feedback on yesterday session* | *All* |
| *0845-0915* | *Theme 3 I help others. Helping others is… (With a ball)* | *Sahar/IK/JFT* |
| *0915-0945* | *Theme 3 I help others. Why and How to help a friend* | *JFT/Sahar/IK* |
| *0945-1015* | *Theme 3 I help others. Who can help?* | *IK/JFT/Sahar* |
| *1015-1030* | *Tea break* |  |
| *1030-1100* | *Theme 4 I understand others: Being a good friend* | *Sahar/IK/JFT* |
| *1100-1130* | *Theme 5: I listen to others. Listen carefully* | *JFT/Sahar/IK* |
| *1130-1200* | *Theme 5: I listen to others Attitudes of a good listener* | *IK/JFT/Sahar* |
| *1200-1230* | *Theme 5: I listen to others. That is what I said. Or no! I did not say that.* | *Sahar/IK/JFT* |
| *1230-1330* | *Lunch break* |  |
| *1330-13-45* | *Different pauses* | *IK/JFT* |
| *1330-1415* | *Theme 6: I listen to myself* | *JFT/Sahar/IK* |
| *1415-1445* | *Theme 6: I listen to myself This is me or not* | *IK/JFT/Sahar* |
| *1445-1500* | *Tea Break* |  |
| *1500-1530* | *Theme 6: I listen to myself What I am good at?* | *Sahar/IK/JFT* |
| *1530-1600* | *Theme 7 I can calm myself Something nice that happened* | *JFT/Sahar/IK* |
| *1600-1615* | *Theme 7 I can calm myself Calming down.* | *IK/JFT/Sahar* |
| *1615-1630* | *Meditations/ Grounding/ Counting breathes from 5 to 0* | *JFT/IK* |
|  | ***Day 4: Wednesday May 18th*** |  |
| *0815-0830* | *Feelings Visualization* | *JFT/IK* |
| *O830-0845* | *Feedback on yesterday session* | *All* |
| *0845-0900* | *Theme 8: I can be assertive confident: Walking assertively, aggressively and passively* | *Sahar/IK/JFT* |
| *0900-0915* | *Theme 8: I can be assertive confident: Personal space* | *JFT/Sahar/IK* |
| *0915-0930* | *Theme 8: I can be assertive confident: Saying no to a friend* | *IK/JFT/Sahar* |
| *0930-1000* | *Theme 9: I can solve conflicts: Asking and saying no* | *Sahar/IK/JFT* |
| *1000-1015* | *Tea break* |  |
| *1015-1045* | *Theme 9: I can solve conflicts: Body language and words when conflicts begin* | *JFT/Sahar/IK* |
| *1045-1115* | *Theme 9: I can solve conflicts: Body language and words to defuse conflicts* | *IK/JFT/Sahar* |
| *1115-1145* | *Theme 9: I can solve conflicts: Stepping up the ladder and stepping down* | *Sahar/IK/JFT* |
| *1145-1215* | *Theme 10: I can cope with change What has changed?* | *JFT/Sahar/IK* |
| *1215-1230* | *Theme 10: I can cope with change Reactions to change* | *IK/JFT/Sahar* |
| *1230-1330* | *Lunch break* |  |
| *1330-13-45* | *Different pauses* | *IK/All* |
| *1330-1400* | *Theme 12 Yes, I can say no: Saying no without saying no, fogging* | *Sahar/IK/JFT* |
| *1400-1415* | *Theme 15 I can move. Jumping Jack* | *JFT/IK* |
| *1415-1445* | *Theme 16 I can relax. Concentrating on an object* | *IK/JFT* |
| *1445-1500* | *Theme 16 I can relax. Shoulders release* | *JFT/IK* |
| *1500-1515* | *Tea break* |  |
| *1515-1530* | *Theme 16 I can relax. Breathing in a color you like* | *JFT/IK* |
| *1530-1600* | *Parents: Activity 1 The Importance of Structure Creating a daily schedule* | *Sahar/IK/JFT* |
| *1600-1615* | *Parents: Activity 1 Start the day with a positive note/ One on one time* | *Sahar/IK/JFT* |
| *1615-1630* | *Calming by awakening the senses* |  |
|  | ***Day 5: Thursday May 19th*** |  |
| *0815-0830* | *Hugging oneself* | *JFT/IK* |
| *O830-0845* | *Feedback on yesterday session* | *All* |
| *0845-0900* | *Activity 2: Promoting Pro-Social Behaviour rather than Negative Discipline. Prosocial behaviors* | *Sahar/IK/JFT* |
| *0900-0915* | *Activity 2: Promoting Pro-Social Behaviour rather than Negative Discipline. Positive discipline* | *Sahar/IK/JFT* |
| *0915-0930* | *Activity 2: Promoting Pro-Social Behaviour rather than Negative Discipline. Conflict resolution* | *Sahar/IK/JFT* |
| *0930-1000* | *Learning through play – creative lessons* | *Sahar/IK/JFT* |
| *1000-1015* | *Tea break* |  |
| *1015-1045* | *Looking after babies and young children at home* | *JFT/IK/Sahar* |
| *1045-1115* | *Communicating with your baby* | *JFT/IK/Sahar* |
| *1115-1145* | *Babies learn through play!* | *IK/JFT/Sahar* |
| *1145-1230* | *Selfcare in difficult times: Strategies* | *Sahar/IK/JFT* |
| *1230-1330* | *Lunch break* |  |
| *1330-1430* | *Fortune Teller game* | *Shamsullah* |
| *1430-1500* | *Dealing with loss and fear of death* | *Sahar/IK/JFT* |
| *1500-1515* | *Tea break* |  |
| *1515-1545* | *Sharing Chores, Dealing with Stigma and Prioritising Self Care* | *Sahar/IK/JFT* |
| *1530-1600* | *Family harmony* | *IK/JFT/Sahar* |
| *1600-1615* | *Reflection and checklist: How is my household doing?* | *JFT/IK/Sahar* |
| *1615-1630* | *Support children to make their hero book* | *Sahar/IK/JFT* |
|  | ***Day 6: Saturday May 20th*** |  |
| *0830-1630* | *Practice in schools* | *All* |
|  | ***Day 7: Sunday May 21st*** |  |
| *0830-1630* | *Practice in schools* | *All* |

1. **Appendix S2. Content of the intervention for teachers and children**
   1. **Teacher Toolkit**

The Teacher Toolkit used in this study is a culturally adapted version of the *“A Hopeful, Healthy, and Happy Living and Learning Toolkit”* developed by the International Federation of the Red Cross and Red Crescent Societies in response to the COVID-19 pandemic. Designed as a psychosocial intervention for teachers and students, it addresses the paucity of culturally sensitive mental health resources in crisis-affected settings such as Afghanistan. Drawing on social-emotional learning (SEL) frameworks, mindfulness-based self-regulation strategies, and evidence-based positive parenting interventions, the toolkit provides structured activities to enhance children’s emotional regulation, social skills, self-awareness, and coping mechanisms for stress and conflict.

Its introduction responds to Afghanistan’s intersecting crises, including regime change, collapsing education and health systems, economic insecurity, exclusion of secondary school girls, and widespread psychosocial distress among children. By combining general exercises with themed activities, the toolkit fosters supportive learning environments, builds resilience, and equips teachers and students with practical strategies to manage anxiety, stress, and interpersonal conflict within resource-limited, high-adversity contexts.

**A. General Exercises**

These activities are designed to create a positive learning environment and equip students and teachers with initial coping mechanisms.

**• A1 Introductory**

- **House** rules **for the classroom**: Encourages collective creation of classroom rules to promote positive social behavior, emphasizing positive phrasing (e.g., "Let one voice be heard at a time," "Respect the opinion of others") and linking rules to values. It also suggests constructive consequences and regular revision of rules, with a focus on restorative consequences for older students.
- **Circle time**: Aims to give every student a voice, foster a sense of belonging, and practice social and emotional skills.
- **Agree with the class on a sign to instruct the class to keep quiet**: Provides non-punitive methods (e.g., raising a hand, "QUIET PLEASE" sign, sibilant sound) for teachers to quiet an excited classroom.

**Yes I made a mistake**: Builds self-confidence by encouraging acceptance of mistakes as part of learning through a counting game where errors are celebrated.

• **A2 Energizers and calming exercises**: Focuses on techniques to help students calm themselves when feeling anxious or stressed.

- **Calming by awakening the senses**: Uses a pencil or bamboo stick to slowly trace the outline of a hand, stimulating skin senses and releasing oxytocin to promote well-being.
- **Breathing with awareness**: Teaches basic breathing exercises (even, deep, calm breaths) to oxygenate the brain and reduce stress.
- **Hugging oneself**: A physical activity to release stress and restore calm by applying firm pressure to the upper arms.
- **Close your eyes and note the sound**: Helps shift focus away from stressful thoughts by paying attention to surrounding sounds.
- **Grounding**: A technique to feel calm and securely rooted by focusing on physical contact with the seat and floor, imagining roots growing from the body.

B. Themed Activities

This section delves into specific psychosocial themes, offering a variety of exercises to develop essential life skills:

• **Theme 1. My school today**: Provides opportunities for students to discuss their school experiences, fostering enjoyment and appreciation for others.

- **Appreciation**: Focuses on identifying and valuing positive traits, qualities, and behaviors in others, which can help reduce irritation in social situations.

• **Theme 2 I know about feelings**: Explores feelings, their causes, and how to manage them.

- **Feelings and why we have them**: Uses a ball game to link feelings to their causes and categorizes them as positive or negative.
- **Identifying important (core) feelings**: Helps students recognize core feelings through silent posture and facial expressions, emphasizing the importance of asking peers how they feel.
- **Wall of feelings**: Encourages expression and recognition of feelings by creating posters for core feelings with associated colors and names, allowing students to visually represent their current mood.
- **X and y get angry, sad, nervous, and afraid: Reasons for feeling**: Examines the reasons behind various feelings through scenarios, promoting understanding of diverse emotional triggers.
- **Changing my mood**: Guides students to identify actions, thoughts, and words that can help change a negative feeling into a positive one.

• **Theme 3: I help others**: Focuses on the act of giving and receiving help, and identifying supportive individuals.

- **Today, I will help…**: Encourages reflection on small and big ways to help others in various settings, highlighting the benefits of kindness such as feeling good, happy, healthy, and calmer.
- **Helping others is… (With a ball)**: Uses a ball game to explore reasons for helping others or needing help, and the feelings associated with receiving help.
- **Why and How to help a friend**: Promotes reflection on how to show care and support to a friend in distress, considering both verbal communication and body language, and acknowledging that help varies by individual.
- **Who can help?**: Helps students identify trusted helpers (e.g., classmates, family, teachers) for various problems, emphasizing that sharing problems reduces stress.

• **Theme 4 I understand others**: Aims to develop understanding of others' thoughts, feelings, and behaviors to foster acceptance and harmonious living.

- **Being a good friend**: Explores qualities of good friendship and how to form new bonds, drawing on characteristics like listening, sympathy, trustworthiness, and support, and integrating Islamic teachings (Hadith) on friendship.

• **Theme 5: I listen to others**: Focuses on developing effective listening skills, acknowledging the verbal and non-verbal factors involved.

- **Listen carefully**: A word association game to practice careful listening and quick responses.
- **Attitudes of a good listener**: Helps students identify and formulate positive attitudes for effective listening, such as patience, non-judgment, full attention, and eye contact.
- **That is what I said. Or no! I did not say that.**: Practices active listening by having students repeat what a partner has said to ensure complete understanding, fostering attentive communication.

• **Theme 6: I listen to myself**: Concentrates on self-awareness by paying attention to physical, emotional, and mental signals.

- **I listen to myself**: Raises awareness of bodily sensations, feelings, and thoughts, and how to act on them. A ball game for younger students differentiates these three domains.
- **This is me or not**: Helps students define their likes, dislikes, and values through a physical game, promoting self-acceptance and understanding of personal boundaries.
- **What I am good at?**: Encourages students to recognize their own capacities and skills and to receive compliments from classmates, fostering self-esteem and acknowledging hidden strengths.

• **Theme 7 I can calm myself**: Focuses on stress management and calming techniques to improve analytical thinking and decision-making in challenging situations.

- **Something nice that happened**: Aims to shift negative feelings by recalling positive memories.
- **Calming down**: Develops a range of physical, social, and cognitive techniques for calming down, including observation exercises, deep breathing, the "STOP" technique for worrying thoughts, and seeking support.
- **Counting breathes from 5 to 0**: A specific breathing exercise designed to induce relaxation and prepare for sleep.

• **Theme 8: I can be assertive confident**: Teaches students how to be assertive, communicate their needs respectfully, negotiate peacefully, and stand up for their rights while respecting others.

- **Walking assertively, aggressively and passively**: Students physically experience and differentiate aggressive, assertive, and submissive behaviors to understand their impact and perception.
- **Personal space**: Defines personal space and its cultural variations, teaching students how to maintain their boundaries and respond to intrusions.
- **Saying no to a friend**: Uses role-playing to practice assertive ways of declining requests in difficult situations, considering factors like gender and age, and linking to real-life scenarios.

• **Theme 9: I can solve conflicts**: Explores conflicts as an inevitable part of life and teaches various strategies for handling them.

- **Asking and saying no**: Practices different ways of asking for something and saying no, focusing on respectful and assertive negotiation techniques.
- **Body language and words when conflicts begin**: Helps students recognize how specific body language and verbal cues can escalate conflicts through scenario-based discussions.
- **Body language and words to defuse conflicts**: Identifies body language and words that help de-escalate conflicts, promoting peaceful resolution.
- **Stepping up the ladder and stepping down**: Raises awareness of the stages of conflict escalation and de-escalation, enabling students to identify steps towards resolution.

• **Theme 10 I can cope with change**: Helps students reflect on change, understand natural reactions to it, and develop strategies for coping, especially with unexpected changes.

- **What has changed?**: A non-verbal observation exercise to practice noticing subtle changes in others, developing observational skills.
- **Reactions to change**: Explores emotional and behavioral reactions to change through a drawing activity where others modify original artwork, leading to discussions on coping mechanisms.

• **Theme 12 Yes, I can say no**: Focuses on appropriate situations to say no, particularly when facing peer pressure, and respectful ways of declining, connecting to personal boundaries and protection.

- **Saying no without saying no, fogging**: Introduces the "fogging" technique as an indirect way to say no and deal with criticism by appearing to agree without engaging in the argument, allowing time to process.

• **Theme 13 I am grateful**: (Listed in Table of Content, p.63 and following, but not detailed in the provided excerpt).

• **Theme 14: I am hopeful**: (Listed in Table of Content, p.67 and following, but not detailed in the provided excerpt).

• **Theme 15 I can move**: Encourages physical activity, stretching, and relaxation in class to improve mental and physical functioning, focus, and concentration, with adaptations for physically challenged students.

- **Jumping Jack and more is a good one we also do here**: Provides opportunities for physical exercise, sharing ideas for movement, and practicing giving clear instructions.

• **Theme 16 I can relax**: Focuses on awareness and concentration exercises to promote relaxation, which can slow heart rate, lower blood pressure, and improve mood and sleep quality.

- **Concentrating on an object**: Teaches concentration as a form of relaxation and a valuable life skill by observing and recalling details of an object.
- **Shoulders release**: Addresses tension in shoulders and neck, common areas of stress, through specific exercises combined with breathing and smiling to release endorphins.
- **Breathing in a color you like**: A deep, restorative breathing exercise where students visualize inhaling a favorite color and exhaling a least favorite color to calm themselves.
  1. **Lifeskills & Mindfulness Activities to do with children**

The second part of the intervention with teachers and students aims to help children and youth develop resilience by building their confidence and sense of purpose through various life skills and mindfulness practices. The intervention is structured into five main sections, each focusing on a different aspect of child development, along with additional mindfulness and yoga activities. It is a comprehensive program that utilizes interactive activities, discussions, and specific mindfulness techniques to foster self-awareness, self-management, social awareness, relationship skills, and responsible decision-making in children, ultimately aiming to build their resilience.

**Section 1: Self-awareness**

This section aims to enhance children's ability to understand their own emotions, thoughts, values, and physical self.

**1** **"Make friends with that feeling"**:

**Aim:** To help children understand the importance of managing strong emotions.

**Activity:** Children (8-12 years old) are asked to describe their current feelings, list common emotions, and then write five strong emotions they've experienced recently on flashcards. They then pick cards and describe the emotion, its cause, and how they handled it.

**Key Concept:** The "NAIL it" system is introduced as a four-step method for managing emotions:

- **N**ame it: Identify the specific feeling (e.g., anger, sadness, fear).
- **A**ccept it: Acknowledge the feeling without self-judgment.
- **I**nvestigate it: Question the emotion's accuracy, appropriateness, and whether it's controlling you, considering its helpfulness and if action is needed.
- **L**et go of it: Act if necessary and calm, but often realize the emotion doesn't require action and can be released to avoid problems.

**Discussion:** Encourages children to recognize recurring strong feelings and create "cute or fun" names for them to foster a friendly relationship with their emotions.

**2 "Believe in me":**

**Aim:** To help children understand the importance of self-belief and maintaining a positive attitude towards life.

**Activity:** Children (8-12 years old) complete a "I am me" handout with prompts like "My best friend is..." and "My father believes that I can...".

**Discussion:** Focuses on developing hope and positivity, being mindful of self-talk, and choosing to be happy and hopeful even when circumstances are challenging.

**Section 2: Self-management**

This section focuses on developing children's ability to manage themselves effectively.

**1 "A heavy bag":**

**Aim:** To help young children understand the importance of talking to someone about their worries.

**Activity:** Children (4-7 years old) navigate a short obstacle course. Initially without a bag, then with increasing weights in a plastic bag. The activity demonstrates how accumulating worries (like heavy objects) makes tasks more difficult.

**Discussion:** Compares the heavy bag to unspoken worries, emphasizing that talking about concerns lightens the burden and identifying trusted individuals to share with.

**2 "If I were a bird":**

**Aim:** To encourage children's imagination, dreams, and hopes, and help them identify practical ways to move toward their goals.

**Activity:** Children (4-7 years old) form pairs and complete "If I were..." sentences (e.g., "If I were a bird, I would...") using pictures or symbols, drawing their answers if they wish. A ball-passing game can facilitate this.

**Discussion:** Encourages reflection on dreams and hopes, and who can assist in realizing them.

**3 "The giant jump rope":**

**Aim:** To focus on the importance of impulse control.

**Activity:** A group of children (8-18 years old) must pass under a long jump rope swung slowly by two adults, without touching it. The rule is they cannot go around the swingers.

**Insight:** Many children impulsively run and touch the rope. The "secret" is to wait, observe, and time the swing, or to go to the end where the person is swinging, requiring careful thought and delayed impulse.

**Discussion:** Relates the activity to real-life situations where thoughtful decision-making is more effective than impulsive actions.

**Section 3: Social awareness**

This section aims to help children develop empathy, understand social norms, and recognize community support by taking others' perspectives.

**1 "Things we have in common":**

**Aim:** To recognize the individuality of each person and commonalities with each other.

**Activity:** Children (8-12 years old) sit in a circle (one less chair than participants). One child states something true about themselves (e.g., "I like the colour red"). Anyone else for whom it's true stands up and walks around with them. When "Stop!" is called, everyone finds a seat, leaving one child to share next.

**Discussion:** Reflects on the experience of discovering shared traits with others in the group.

**2 "Cross the line":**

**Aim:** To help children understand the basics and importance of empathy.

**Activity:** In complete silence, children (8-12 years old) stand behind a line. The facilitator calls out various life experiences (e.g., "the death of the grandpa"). If an experience is true for a child, they cross the line and turn to face others, with an option to stay put if uncomfortable. After a pause, everyone returns behind the line.

**Discussion:** Focuses on the feelings evoked by crossing the line or watching others, learning about oneself and others. Empathy is defined as "walking in someone else's shoes for 10 kilometers" and is presented as a key skill for navigating a diverse world, preventing judgment, hatred, violence, discrimination, and isolation. Participants are encouraged to practice empathy.

**3 "Ball of string":**

**Aim:** To encourage inclusion and demonstrate how everyone is connected.

**Activity:** Children (4-7 years old) stand in a circle. One person holds a ball of string, states something they can give to others (e.g., "a smile"), and then rolls the ball to someone else while still holding onto their part of the string. This continues until a "web" of connections is formed. Then, a few children drop their string, illustrating how the web weakens.

**Discussion:** Emphasizes that exclusion weakens the group and highlights everyone's role in supporting each other.

**Section 4: Relationship skills**

This section aims to help children build skills for making and maintaining healthy and positive relationships, including communication, negotiation, resisting negative peer pressure, and conflict resolution.

**1 "I am a good listener when…":**

**Aim:** To identify key listening skills.

**Activity:** Small groups (8-12 years old) brainstorm and write down ideas that complete the statement "I am a good listener when..." Examples include: "Do not interrupt," "Listen carefully," "Concentrate on what is being said," "Look towards the person talking," "Keep quiet," "I do not judge," "I empathize," and "I have eye contact".

**Discussion:** Encourages self-reflection on listening habits, when interrupting is acceptable, and identifying people they want to listen to more, or who they want to listen to them more.

**2 "Teamwork":**

**Aim:** To focus children on the benefits of collaboration and teamwork.

**Activity:** Children (8-12 years old) work in pairs to transport an object through an obstacle course without using their hands, instead using various body parts (e.g., elbow to elbow, chin to chin, back to back). Each pair must use a different combination of body parts. If the object is dropped, they restart. Finally, the whole team carries an object, all in contact with it.

**Discussion:** Reflects on the challenges of not using hands, the ease of cooperation, and how teamwork can make tasks easier and more fun at home, school, or with friends.

**3 "Balloon in a bottle":**

**Aim:** To help children understand and resist peer pressure.

**Activity:** Children (8-12 years old) attempt to inflate a deflated balloon inside a bottle labeled "Peer Pressure." They discover it's impossible. They then inflate it outside the bottle.

**Discussion:** The balloon represents the child, and the bottle represents peer pressure. The activity illustrates that peer pressure "will not allow you to become your own person" and can "stop you from growing". Strategies to avoid peer pressure are discussed, such as spending time with positive influences, learning to say no, asking for help, and walking away from bad situations.

**4 "The wise owl":**

**Aim:** To explore different conflict resolution styles and encourage effective negotiation.

**Activity:** Children role-play resolving disagreements, each adopting the style of an animal archetype: the **tortoise** (withdraws), the **teddy bear** (gives in to create peace), the **shark** (fights to get their own way), or the **owl** (tries to find a mutually agreeable solution).

**Discussion:** Children reflect on how each animal role feels and identify with their own typical conflict style. Tips for being an "owl" include listening carefully, showing respect for others' ideas, not shouting, encouraging others to share, asking "sharks" to cool down, and finding common ground while maintaining friendships.

**Section 5: Responsible Decision-making**

This section introduces children to an effective method for making wise choices.

**1 "Stranded on a desert or in the mountain":**

**Aim:** To introduce an effective way of making wise choices.

**Activity:** Small groups of children (8-12 years old) imagine being stranded with a broken car and limited items. They choose one item each, explaining their reasoning, and then brainstorm how to improve their chances of survival by creatively combining items or convincing others to bring different ones.

**Discussion:** The activity highlights that life presents important decisions, and many people struggle with decision-making by avoiding it or rushing, leading to negative consequences.

**Key Concept:**

The **"STEP System"** is introduced as a simple recipe for making wise choices:

**S**ay it out loud: Verbalize or write down the decision to make it real and build accountability.

**T**hink about your options: Brainstorm all possible courses of action.

**E**xplore the consequences: For each option, consider what is likely to happen, being honest and accurate about the outcomes. This critical step is often skipped, leading to problems.

**P**ick one: Choose the best option and commit to it, letting go of other choices. If the chosen path doesn't work, one can always return to these steps to rethink the process.

Mindfulness and Yoga Activities

These additional activities aim to promote physical and emotional relaxation and focus.

**2 Progressive Muscle Relaxation for Children:**

**Purpose:** To generate physical and emotional relaxation, reduce tension, stress, and anxiety, and aid sleep.

**Techniques:** Involves tensing and then relaxing different muscle groups throughout the body, combined with deep breathing. This includes focusing on legs and feet, hands and arms, stomach, arms and shoulders, shoulders and neck, and face, often with imaginative scenarios (e.g., squeezing an orange, shielding from rain).

**3 Yoga and Mindfulness Games and Group Activities:**

**"Slow motion fast forward"**: Teaches sun salutations (or half) at very slow or super speedy paces. This helps children (using jumping jacks + mountain pose) recognize shifts in their physical body, breath, and heartbeat.

**"Evolution"**: Participants in a circle pass along yoga postures or movements. A leader changes the posture when it returns to them. Balancing poses like Warrior 2, airplane, tree, and eagle are used, sometimes with a focus object like a stone or marble.

**"If It’s True for You"**: A leader reads statements (e.g., "I like dogs"). If true for a participant, they perform a specific yoga pose (e.g., tree pose); if not, they hold a different pose (e.g., mountain pose).

**4 Meditations:**

**Breathe Meditation/Anchor Breathing**: Children lie or sit comfortably, focus on long, slow, deep breaths, imagining their breath as an anchor that keeps them steady and connected to inner calmness, especially when their mind is chaotic or stressed.

**Magic Carpet**: Children lie down and visualize themselves resting and flying on a magic carpet, paying attention to sensory details like colors, texture, and what they see, hear, smell, and feel. This exercise enhances sensory engagement and relaxation.

**Feelings Visualization**: Children practice deep belly breathing and visualize a stressful emotion (e.g., anger, fear) as a distinct shape, color, size, and texture. They observe it as separate from themselves and, when ready, can let it drift away. This teaches them to recognize and manage emotions without being controlled by them. Other techniques like "Lion's Breath" (strong exhale) and nostril exercises are also mentioned.

1. **Appendix S3. Content of the intervention for parents**

We culturally adapted the "Parents & Caregivers Toolkit: A Hopeful, Healthy and Happy Living and Learning Toolkit". It serves as a guide for parents and caregivers, offering activities and principles to foster child resilience, positive behavior, and well-being within the family, especially in challenging times. It is composed of ten activities.

1. **The Importance of Structure**

This section emphasizes the value of creating a daily schedule for families, including structured activities, chores, fun, and free time. Involving children in schedule creation helps them follow it better, and predictability provides security for children and reduces stress for parents. The schedule should include physical exercise daily to help with stress and energy levels. Special attention is given to children with special needs, for whom routine changes can be confusing, highlighting the importance of a comfortable and predictable environment. The toolkit also advises parents to start the day with a positive note, encouraging family breakfasts, asking about feelings, promoting empathy, and setting a positive tone for the day. One-on-one time with children is highlighted as crucial for listening, supporting, and reassuring them, allowing children to choose activities or topics of discussion.

1. **Promoting Pro-Social Behaviour rather than Negative Discipline**

This section differentiates between negative and positive discipline, advocating for the latter, which focuses on promoting desired behaviors rather than punishing undesired ones. Parents are encouraged to manage their anger, take deep breaths, and communicate desired behavior calmly. The document lists several pro-social behaviors and psychosocial skills to encourage, such as self-awareness, empathy, goal setting, impulse control, communication, and conflict handling. Positive discipline involves helping children learn appropriate ways to handle situations while remaining calm, friendly, and respectful. It explicitly recommends against negative discipline, which can involve angry, destructive, or violent responses, as it can teach children violence and damage trust.

1. **Conflict Resolution**

When conflicts arise, the first step is to help children calm down by approaching them calmly, at eye level, with gentle touch, and suggesting breathing exercises. Adults should stay neutral, avoid blame, and "contain" emotionally charged situations by being the calm presence. The guide suggests talking about wants and needs, focusing on what each child wants rather than what happened, and acknowledging their feelings. The problem should be defined neutrally, and children should be helped to brainstorm fair solutions themselves to develop their problem-solving skills.

1. **Learning through play – creative lessons**

This section encourages engaging children's interest and focus through games and group activities for learning. Examples include using cooking for math practice, explaining science through nature, and reading stories.

1. **Looking after babies and young children at home**

This part addresses the demands of caring for very young children, especially during a crisis. It notes that young children are highly dependent on adult care and can be overwhelmed by caregivers' anxiety, which may lead to regressed behaviors like clinginess or changes in eating/sleeping habits. It provides ideas for communicating with babies, such as mirroring their actions, repeating babbling, using their name, describing their actions, and engaging in back-and-forth interactions. It also emphasizes that babies learn through play, suggesting activities like peek-a-boo, singing, making noises with objects, and sharing books.

1. **Self-care in difficult times**

Five strategies are outlined for self-care:

1. Strategy 1: Daily routine – Maintain a routine, take regular breaks, eat/drink regularly, avoid unhelpful coping strategies like smoking or opium, limit news consumption, and get sufficient sleep (at least seven hours).
2. Strategy 2: Exercise – Plan regular exercise, such as walks or runs, play games, and practice breathing exercises to reduce stress.
3. Strategy 3: Stay connected – Maintain spiritual practices and stay in touch with family and friends.
4. Strategy 4: Accept your feelings – Recognize that stress, anger, and other emotions are normal responses to challenging situations, and it's okay to not be okay and to seek support.
5. Strategy 5: Stay hopeful – Recall past coping strategies, focus on what's in control, celebrate small wins, and view challenges as a "marathon" that will eventually improve.
6. **Dealing with loss and fear of death**

This section advises against avoiding conversations about loss with children, emphasizing that children need to grieve and remember those who died to release painful feelings and build positive memories. Caregivers should be aware of children's specific losses (e.g., death, loss of normal routines) and their potential anxiety about current caregivers dying. It's important to reassure children that most people recover from illnesses like COVID-19 and that treatment exists. Common grief reactions are listed, including shock, anxiety, anger, sadness, and changes in sleep or appetite.

1. **Multiple intelligence – the many ways we can be clever**

The toolkit promotes building children's confidence by praising their strengths and different "multiple intelligences". It encourages parents to identify their children's natural intelligences and create opportunities for them to succeed in these areas, also praising consideration towards others as a form of emotional and social intelligence. The theory of multiple intelligences is explained, listing nine types: Visual-spatial, Mathematical-logical, Body, Verbal, Musical-dance, Emotional, Social, Naturalistic, and Philosophical-spiritual intelligence.

1. **Sharing Chores, Dealing with Stigma and Prioritising Self Care**

This section encourages sharing household chores, childcare, and tasks equally among family members and integrating them into the weekly schedule, suggesting making cleaning a game. It defines stigma as holding negative attitudes against identifiable groups, often fueled by lack of knowledge, fear, and gossip. Stigma can lead to labeling, discrimination, violence, and prevent people from seeking help, ultimately making it harder to control disease outbreaks. Prioritizing self-care is crucial for caregivers to manage their own anxiety, anger, and frustrations. Ideas for self-care include prioritizing wellbeing (eating, exercise, sleep), planning relaxing activities, taking time to daydream, staying socially connected, setting realistic goals, injecting humor, maintaining hope, and using stress management techniques. Parents are also advised to reach out to teachers and other parents for support and advice.

1. **End the day on a good note**

Parents are encouraged to end the day positively by reinforcing good behaviors, celebrating successes, checking in with each family member about their feelings, worries, gratitude, and hopes for the future.

| Table S1. Comparison between children who stayed and dropped out | | | |
| --- | --- | --- | --- |
|  | Dropout (N=617) | Stay (N=3210) | t Test/Chi2 p value |
| Age |  |  |  |
| Mean (SD) | 11.7 (1.84) | 11.5 (1.48) | t =-1.88, p=0.06 |
| Median [Min, Max] | 12.0 [7.00, 16.0] | 12.0 [7.00, 16.0] |  |
| Ethnicity |  |  |  |
| Tajik | 25 (4.1%) | 605 (18.8%) | F = 0.44. p=0.64 |
| Pashtun | 408 (66.1%) | 1166 (36.3%) |  |
| Other | 184 (29.8%) | 1439 (44.8%) |  |
| Sex |  |  |  |
| Male | 261 (42.3%) | 1387 (43.2%) | F = 0.06. p=0.80 |
| Female | 356 (57.7%) | 1823 (56.8%) |  |
| Grade |  |  |  |
| 3 | 183 (29.7%) | 382 (11.9%) | F = 1.13. p=0.32 |
| 4 | 48 (7.8%) | 774 (24.1%) |  |
| 5 | 180 (29.2%) | 1025 (31.9%) |  |
| 6 | 206 (33.4%) | 1029 (32.1%) |  |
| Area |  |  |  |
| Ghazni | 20 (3.2%) | 963 (30.0%) | F =0.04. p=0.99 |
| Jaghori | 122 (19.8%) | 517 (16.1%) |  |
| Takhar | 30 (4.9%) | 1008 (31.4%) |  |
| Badakhshan | 445 (72.1%) | 722 (22.5%) |  |
| Assets |  |  |  |
| Mean (SD) | -0.351 (1.71) | 0.0256 (1.77) | t =-1.89, p=0.06 |
| Median [Min, Max] | -0.716 [-3.33, 9.18] | -0.309 [-3.37, 11.1] |  |
| Missing | 110 (17.8%) | 30 (0.9%) |  |
| Livestock |  |  |  |
| Mean (SD) | 0.123 (1.21) | 0.123 (1.28) | t =-0.002, p=0.99 |
| Median [Min, Max] | 0.450 [-4.99, 1.75] | 0.432 [-10.4, 1.89] |  |
| Missing | 110 (17.8%) | 30 (0.9%) |  |
| Life skills | | | |
| Mean (SD) | 46.0 (12.3) | 53.9 (12.8) | t =4.11, p<0.001 |
| Median [Min, Max] | 43.0 [15.8, 76.0] | 53.0 [0, 76.0] |  |
| Resilience |  |  |  |
| Mean (SD) | 40.5 (4.99) | 40.3 (5.00) | t =-0.62, p=0.54 |
| Median [Min, Max] | 42.0 [20.0, 44.0] | 42.0 [2.00, 44.0] |  |
| Self-efficacy |  |  |  |
| Mean (SD) | 45.4 (12.4) | 51.3 (13.4) | t =4.11, p<0.001 |
| Median [Min, Max] | 43.0 [15.0, 80.0] | 50.0 [0, 80.0] |  |
| Discrimination |  |  |  |
| Mean (SD) | 1.23 (2.42) | 0.915 (1.81) | t =-0.88, p=0.38 |
| Median [Min, Max] | 0 [0, 12.0] | 0 [0, 16.0] |  |
| Stigma |  |  |  |
| Mean (SD) | 4.65 (2.95) | 2.89 (2.58) | t =-5.81, p<0.001 |
| Median [Min, Max] | 4.00 [0, 20.0] | 2.00 [0, 20.0] |  |
| Anxiety |  |  |  |
| Mean (SD) | 10.7 (3.65) | 9.22 (4.36) | t =-5.07, p<0.001 |
| Median [Min, Max] | 11.0 [0, 24.0] | 6.00 [0, 24.0] |  |
| Depression |  |  |  |
| Mean (SD) | 15.6 (5.68) | 14.2 (6.69) | t =-3.37, p=0.001 |
| Median [Min, Max] | 16.0 [10.0, 40.0] | 10.0 [0, 42.0] |  |
| Reading literacy |  |  |  |
| Mean (SD) | 2.51 (1.34) | 2.68 (1.32) | t =1.40, p=0.16 |
| Median [Min, Max] | 2.00 [0, 4.00] | 3.00 [0, 4.00] |  |
| Mathematical literacy |  |  |  |
| Mean (SD) | 2.26 (0.853) | 2.56 (0.921) | t =3.86, p<0.001 |
| Median [Min, Max] | 2.00 [0, 4.00] | 2.00 [0, 4.00] |  |
| Mathematical problem solving |  |  |  |
| Mean (SD) | 2.16 (0.667) | 2.31 (0.741) | t=2.93, p=0.004 |
| Median [Min, Max] | 2.00 [0, 3.00] | 2.00 [0, 3.00] |  |

Table S2. Effects of intervention length on child mental well-being outcomes, school-based stigma and discrimination, depression, anxiety, and academic outcomes including covariates

|  | Shorter Intervention (Ref: Control Schools) | | | | Longer Intervention (Ref: Control Schools) | | | | Wave | | | | Shorter Intervention x Wave | | | | Longer Intervention x Wave | | | |
| --- | --- | --- | --- | --- | --- | --- | --- | --- | --- | --- | --- | --- | --- | --- | --- | --- | --- | --- | --- | --- |
| *Outcomes* | *Estimates* | *CI* | *p* | *Estimates* | | *CI* | *p* | *Estimates* | | *CI* | *p* | *Estimates* | | *CI* | *p* | *Estimates* | | *CI* | *p* |  |
| Life skills | 0.03 | -0.12 –0.18 | 0.710 | 0.48 | | 0.36 – 0.61 | **<0.001** | 0.07 | | 0.02 – 0.12 | **0.003** | 0.11 | | 0.01 – 0.20 | **0.036** | -0.30 | | -0.38 –-0.22 | **<0.001** |  |
| Resilience | 0.03 | -0.13-0.18 | 0.744 | 0.10 | | -0.03 –0.22 | 0.135 | 0.07 | | 0.02 – 0.12 | **0.006** | -0.04 | | -0.14 –0.06 | 0.444 | -0.04 | | -0.12 –0.05 | 0.400 |  |
| Self-Efficacy | 0.004 | -0.16 –0.17 | 0.960 | 0.21 | | 0.08 –0.35 | **0.002** | 0.03 | | -0.03 –0.08 | 0.358 | 0.08 | | -0.02 –0.19 | 0.124 | -0.13 | | -0.22 –-0.04 | **0.003** |  |
| Stigma | 0.57 | 0.39 – 0.75 | **<0.001** | -0.46 | | -0.61 –-0.31 | **<0.001** | -0.21 | | -0.27 –-0.15 | **<0.001** | -0.34 | | -0.46 –-0.22 | **<0.001** | 0.34 | | 0.24 –0.44 | **<0.001** |  |
| Discrimination | 0.49 | 0.29 –0.68 | **<0.001** | 0.35 | | 0.19 –0.51 | **<0.001** | -0.18 | | -0.25 –-0.12 | **<0.001** | -0.19 | | -0.31 –-0.06 | **0.003** | -0.20 | | -0.31 –-0.10 | **<0.001** |  |
| Depression | 0.18 | 0.001 –0.37 | **0.048** | -0.23 | | -0.39 –-0.08 | **0.003** | -0.26 | | -0.31 –-0.20 | **<0.001** | -0.18 | | -0.30 –-0.06 | **0.003** | 0.10 | | -0.001– 0.20 | 0.051 |  |
| Anxiety | 0.30 | 0.12 –0.48 | **0.001** | -0.31 | | -0.47 –-0.16 | **<0.001** | -0.24 | | -0.30 – -0.18 | **<0.001** | -0.24 | | -0.36 –-0.12 | **<0.001** | 0.17 | | 0.07 – 0.27 | **0.001** |  |
| Reading/general knowledge | -0.26 | -0.40 –-0.12 | **<0.001** | -0.41 | | -0.53 –-0.29 | **<0.001** | 0.19 | | 0.14 – 0.23 | **<0.001** | 0.15 | | 0.06 – 0.23 | **0.001** | 0.27 | | 0.20 – 0.34 | **<0.001** |  |
| Numeracy | -0.27 | -0.41 –-0.13 | **<0.001** | -0.31 | | -0.42 –-0.19 | **<0.001** | 0.20 | | 0.16 – 0.25 | **<0.001** | 0.17 | | 0.08 – 0.26 | **<0.001** | 0.20 | | 0.13 – 0.27 | **<0.001** |  |
| Arithmetic problem solving | -0.18 | -0.34 – -0.02 | **0.027** | -0.10 | | -0.24 – 0.03 | 0.137 | 0.09 | | 0.04 – 0.15 | **<0.001** | 0.14 | | 0.03 – 0.24 | **0.010** | 0.05 | | -0.03 – 0.14 | 0.242 |  |

Note: Standard errors are clustered at the school level. Age, gender, ethnicity, district, assets, and livestock were used as control variables.

Table S3. Effects of intervention on girls’ mental well-being outcomes, school-based stigma and discrimination, depression, anxiety, and academic outcomes, including covariates

|  | Intervention | | | Wave | | | Intervention × Wave | | |
| --- | --- | --- | --- | --- | --- | --- | --- | --- | --- |
| *Outcomes* | *Estimates* | *CI* | *p* | *Estimates* | *CI* | *p* | *Estimates* | *CI* | *p* |
| Life skills | 0.48 | 0.31 – 0.64 | **<0.001** | 0.12 | 0.05 – 0.20 | **0.001** | -0.23 | -0.33 – -0.12 | **<0.001** |
| Resilience | -0.01 | -0.20 – 0.18 | 0.932 | 0.11 | 0.03 – 0.20 | **0.011** | 0.03 | -0.10 – 0.15 | 0.683 |
| Self-Efficacy | 0.26 | 0.08 – 0.44 | **0.004** | 0.03 | -0.05 – 0.11 | 0.403 | -0.11 | -0.22 – 0.01 | 0.066 |
| Stigma | -0.26 | -0.44 – -0.08 | **0.004** | -0.23 | -0.32 – -0.15 | **<0.001** | 0.17 | 0.05 – 0.28 | **0.005** |
| Discrimination | 0.22 | 0.03 – 0.41 | **0.021** | -0.20 | -0.29 – -0.12 | **<0.001** | -0.10 | -0.23 – 0.02 | 0.092 |
| Depression | -0.32 | -0.51 – -0.14 | **<0.001** | -0.34 | -0.42 – -0.26 | **<0.001** | 0.13 | 0.02 – 0.25 | **0.027** |
| Anxiety | -0.32 | -0.50 – -0.14 | **0.001** | -0.31 | -0.39 – -0.22 | **<0.001** | 0.14 | 0.03 – 0.26 | **0.017** |
| Reading/general knowledge | -0.43 | -0.58 – -0.29 | **<0.001** | 0.19 | 0.13 – 0.25 | **<0.001** | 0.22 | 0.13 – 0.30 | **<0.001** |
| Numeracy | -0.25 | -0.40 – -0.11 | **0.001** | 0.26 | 0.20 – 0.33 | **<0.001** | 0.11 | 0.02 – 0.20 | **0.014** |
| Arithmetic problem solving | -0.02 | -0.19 – 0.15 | 0.829 | 0.18 | 0.11 – 0.26 | **<0.001** | -0.02 | -0.13 – 0.09 | 0.716 |

Note: Standard errors are clustered at the school level. Age, ethnicity, district, assets, and livestock were used as control variables.

Table S4. Effects of intervention length on girls’ mental well-being outcomes, school-based stigma and discrimination, depression, anxiety, and academic outcomes, including covariates

|  | Shorter Intervention (Ref: Control Schools) | | | Longer Intervention (Ref: Control Schools) | | | Wave | | | Shorter Intervention x Wave | | | Longer Intervention x Wave | | |
| --- | --- | --- | --- | --- | --- | --- | --- | --- | --- | --- | --- | --- | --- | --- | --- |
| *Outcomes* | *Estimates* | *CI* | *p* | *Estimates* | *CI* | *p* | *Estimates* | *CI* | *p* | *Estimates* | *CI* | *p* | *Estimates* | *CI* | *p* |
| Life skills | 0.27 | 0.05 – 0.49 | **0.018** | 0.72 | 0.53 – 0.92 | **<0.001** | 0.15 | 0.07 – 0.22 | **<0.001** | -0.08 | -0.23 – 0.06 | 0.263 | -0.42 | -0.54 – -0.30 | **<0.001** |
| Resilience | -0.23 | -0.49 – 0.03 | 0.084 | 0.12 | -0.10 – 0.34 | 0.298 | 0.12 | 0.03 – 0.20 | **0.008** | 0.10 | -0.07 – 0.27 | 0.234 | -0.03 | -0.18 – 0.11 | 0.674 |
| Self-Efficacy | 0.27 | 0.03 – 0.51 | **0.030** | 0.36 | 0.15 – 0.56 | **0.001** | 0.06 | -0.02 – 0.14 | 0.143 | -0.12 | -0.27 – 0.04 | 0.132 | -0.19 | -0.32 – -0.06 | **0.004** |
| Stigma | 0.17 | -0.08 – 0.41 | 0.176 | -0.55 | -0.76 – -0.34 | **<0.001** | -0.24 | -0.32 – -0.16 | **<0.001** | -0.10 | -0.26 – 0.05 | 0.193 | 0.37 | 0.24 – 0.51 | **<0.001** |
| Discrimination | 0.37 | 0.12 – 0.63 | **0.004** | 0.37 | 0.15 – 0.59 | **0.001** | -0.16 | -0.25 – -0.08 | **<0.001** | -0.16 | -0.33 – 0.00 | 0.057 | -0.22 | -0.36 – -0.08 | **0.003** |
| Depression | -0.05 | -0.29 – 0.20 | 0.710 | -0.41 | -0.62 – -0.19 | **<0.001** | -0.32 | -0.40 – -0.24 | **<0.001** | -0.05 | -0.21 – 0.11 | 0.539 | 0.19 | 0.05 – 0.32 | **0.008** |
| Anxiety | -0.07 | -0.32 – 0.17 | 0.560 | -0.45 | -0.66 – -0.24 | **<0.001** | -0.30 | -0.38 – -0.22 | **<0.001** | -0.03 | -0.19 – 0.13 | 0.690 | 0.24 | 0.11 – 0.38 | **<0.001** |
| Reading/general knowledge | -0.48 | -0.68 – -0.28 | **<0.001** | -0.51 | -0.67 – -0.34 | **<0.001** | 0.19 | 0.13 – 0.25 | **<0.001** | 0.15 | 0.03 – 0.27 | **0.011** | 0.27 | 0.17 – 0.37 | **<0.001** |
| Numeracy | -0.32 | -0.52 – -0.12 | **0.002** | -0.23 | -0.40 – -0.06 | **0.008** | 0.28 | 0.21 – 0.34 | **<0.001** | 0.09 | -0.03 – 0.21 | 0.158 | 0.09 | -0.01 – 0.19 | 0.085 |
| Arithmetic problem solving | -0.10 | -0.33 – 0.14 | 0.417 | 0.01 | -0.19 – 0.21 | 0.934 | 0.19 | 0.11 – 0.26 | **<0.001** | 0.02 | -0.12 – 0.17 | 0.768 | -0.05 | -0.18 – 0.07 | 0.390 |

Note: Standard errors are clustered at the school level. Age, ethnicity, district, assets, and livestock were used as control variables.

Table S5. Effects of intervention on boys’ mental well-being outcomes, school-based stigma and discrimination, depression, anxiety, and academic outcomes, including covariates

|  | Intervention | | | Wave | | | Intervention × Wave | | |
| --- | --- | --- | --- | --- | --- | --- | --- | --- | --- |
| *Outcomes* | *Estimates* | *CI* | *p* | *Estimates* | *CI* | *p* | *Estimates* | *CI* | *p* |
| Life skills | 0.01 | -0.17 – 0.20 | 0.899 | -0.02 | -0.11 – 0.06 | 0.581 | 0.04 | -0.08 – 0.16 | 0.558 |
| Resilience | 0.22 | 0.01 – 0.44 | **0.045** | 0.06 | -0.04 – 0.16 | 0.227 | -0.13 | -0.27 – 0.02 | 0.082 |
| Self-Efficacy | -0.13 | -0.32 – 0.07 | 0.200 | -0.03 | -0.12 – 0.06 | 0.496 | 0.11 | -0.02 – 0.23 | 0.088 |
| Stigma | 0.30 | 0.10 – 0.50 | **0.003** | -0.14 | -0.23 – -0.04 | **0.005** | -0.11 | -0.24 – 0.02 | 0.108 |
| Discrimination | 0.47 | 0.26 – 0.68 | **<0.001** | -0.20 | -0.29 – -0.10 | **<0.001** | -0.21 | -0.34 – -0.07 | **0.002** |
| Depression | 0.25 | 0.04 – 0.45 | **0.019** | -0.15 | -0.25 – -0.06 | **0.002** | -0.18 | -0.32 – -0.05 | **0.007** |
| Anxiety | 0.30 | 0.09 – 0.51 | **0.005** | -0.13 | -0.23 – -0.03 | **0.008** | -0.19 | -0.33 – -0.06 | **0.005** |
| Reading/general knowledge | -0.21 | -0.37 – -0.05 | **0.008** | 0.19 | 0.12 – 0.25 | **<0.001** | 0.25 | 0.15 – 0.34 | **<0.001** |
| Numeracy | -0.40 | -0.56 – -0.24 | **<0.001** | 0.11 | 0.04 – 0.18 | **0.003** | 0.36 | 0.26 – 0.46 | **<0.001** |
| Arithmetic problem solving | -0.31 | -0.50 – -0.13 | **0.001** | -0.03 | -0.12 – 0.05 | 0.480 | 0.26 | 0.14 – 0.38 | **<0.001** |

Note: Standard errors are clustered at the school level. Age, ethnicity, district, assets, and livestock were used as control variables.

Table S6. Effects of intervention length on boys’ mental well-being outcomes, school-based stigma and discrimination, depression, anxiety, and academic outcomes, including covariates

|  | Shorter Intervention (Ref: Control Schools) | | | Longer Intervention (Ref: Control Schools) | | | Wave | | | Shorter Intervention x Wave | | | Longer Intervention x Wave | | |
| --- | --- | --- | --- | --- | --- | --- | --- | --- | --- | --- | --- | --- | --- | --- | --- |
| *Outcomes* | *Estimates* | *CI* | *p* | *Estimates* | *CI* | *p* | *Estimates* | *CI* | *p* | *Estimates* | *CI* | *p* | *Estimates* | *CI* | *p* |
| Life skills | -0.31 | -0.57 – -0.06 | **0.017** | 0.29 | 0.08 – 0.50 | **0.007** | -0.01 | -0.09 – 0.07 | 0.830 | 0.39 | 0.23 – 0.56 | **<0.001** | -0.20 | -0.34 – -0.07 | **0.003** |
| Resilience | 0.37 | 0.06 – 0.67 | **0.018** | 0.14 | -0.11 – 0.39 | 0.270 | 0.06 | -0.04 – 0.16 | 0.256 | -0.24 | -0.44 – -0.04 | **0.016** | -0.06 | -0.22 – 0.10 | 0.475 |
| Self-Efficacy | -0.35 | -0.62 – -0.08 | **0.012** | 0.07 | -0.15 – 0.29 | 0.525 | -0.02 | -0.11 – 0.07 | 0.692 | 0.37 | 0.20 – 0.54 | **<0.001** | -0.07 | -0.21 – 0.07 | 0.332 |
| Stigma | 1.07 | 0.80 – 1.35 | **<0.001** | -0.35 | -0.57 – -0.12 | **0.003** | -0.18 | -0.27 – -0.09 | **<0.001** | -0.64 | -0.82 – -0.46 | **<0.001** | 0.29 | 0.15 – 0.44 | **<0.001** |
| Discrimination | 0.57 | 0.28 – 0.86 | **<0.001** | 0.36 | 0.12 – 0.59 | **0.003** | -0.21 | -0.31 – -0.11 | **<0.001** | -0.21 | -0.40 – -0.02 | **0.028** | -0.19 | -0.35 – -0.04 | **0.014** |
| Depression | 0.56 | 0.27 – 0.84 | **<0.001** | -0.03 | -0.27 – 0.20 | 0.772 | -0.18 | -0.27 – -0.08 | **<0.001** | -0.38 | -0.57 – -0.20 | **<0.001** | -0.02 | -0.17 – 0.13 | 0.828 |
| Anxiety | 0.86 | 0.57 – 1.15 | **<0.001** | -0.16 | -0.40 – 0.08 | 0.187 | -0.16 | -0.25 – -0.07 | **0.001** | -0.55 | -0.73 – -0.36 | **<0.001** | 0.07 | -0.08 – 0.22 | 0.356 |
| Reading/general knowledge | 0.01 | -0.22 – 0.23 | 0.960 | -0.32 | -0.50 – -0.14 | **0.001** | 0.19 | 0.12 – 0.26 | **<0.001** | 0.15 | 0.02 – 0.28 | **0.027** | 0.29 | 0.18 – 0.39 | **<0.001** |
| Numeracy | -0.24 | -0.47 – -0.02 | **0.036** | -0.43 | -0.61 – -0.25 | **<0.001** | 0.12 | 0.05 – 0.19 | **0.001** | 0.30 | 0.16 – 0.44 | **<0.001** | 0.36 | 0.25 – 0.47 | **<0.001** |
| Arithmetic problem solving | -0.31 | -0.57 – -0.05 | **0.019** | -0.27 | -0.48 – -0.05 | **0.014** | -0.02 | -0.10 – 0.07 | 0.705 | 0.31 | 0.14 – 0.47 | **<0.001** | 0.19 | 0.06 – 0.33 | **0.004** |

Note: Standard errors are clustered at the school level. Age, ethnicity, district, assets, and livestock were used as control variables.

Table S7a. Effects of intervention on child mental well-being outcomes, school-based stigma and discrimination, depression, anxiety, and academic outcomes, including covariates in Badakhshan

|  | Intervention | | | Wave | | | Intervention × Wave | | |
| --- | --- | --- | --- | --- | --- | --- | --- | --- | --- |
| *Outcomes* | *Estimates* | *CI* | *p* | *Estimates* | *CI* | *p* | *Estimates* | *CI* | *p* |
| Life skills | -0.82 | -1.07 – -0.56 | **<0.001** | -0.003 | -0.13 – 0.12 | 0.964 | 0.65 | 0.48 – 0.82 | **<0.001** |
| Resilience | 0.05 | -0.23 – 0.33 | 0.722 | -0.10 | -0.24 – 0.036 | 0.147 | 0.03 | -0.15 – 0.22 | 0.717 |
| Self-Efficacy | -1.02 | -1.27 – -0.76 | **<0.001** | 0.03 | -0.10 – 0.15 | 0.676 | 0.70 | 0.53 – 0.87 | **<0.001** |
| Stigma | 0.77 | 0.51 – 1.03 | **<0.001** | -0.35 | -0.48 – -0.22 | **<0.001** | -0.46 | -0.64 – -0.28 | **<0.001** |
| Discrimination | 0.49 | 0.22 – 0.77 | **<0.001** | -0.43 | -0.57 – -0.30 | **<0.001** | -0.19 | -0.38 – -0.004 | **0.045** |
| Depression | 0.34 | 0.05 – 0.62 | **0.020** | -0.11 | -0.25 – 0.03 | 0.138 | -0.13 | -0.32 – 0.06 | 0.172 |
| Anxiety | 0.54 | 0.27 – 0.82 | **<0.001** | -0.28 | -0.41 – -0.14 | **<0.001** | -0.26 | -0.44 – -0.08 | **0.006** |
| Reading/general knowledge | -0.40 | -0.61 – -0.19 | **<0.001** | 0.17 | 0.08 – 0.27 | **0.001** | 0.39 | 0.26 – 0.53 | **<0.001** |
| Numeracy | -0.59 | -0.81 – -0.37 | **<0.001** | 0.22 | 0.11 – 0.33 | **<0.001** | 0.49 | 0.35 – 0.64 | **<0.001** |
| Arithmetic problem solving | -0.46 | -0.71 – -0.20 | **<0.001** | -0.16 | -0.29 – -0.04 | **0.012** | 0.42 | 0.25 – 0.59 | **<0.001** |

Note: Standard errors are clustered at the school level. Age, gender, ethnicity, assets, and livestock were used as control variables.

Table S7b. Effects of intervention length on child mental well-being outcomes, school-based stigma and discrimination, depression, anxiety, and academic outcomes, including covariates in Badakhshan

|  | Shorter Intervention (Ref: Control Schools) | | | Longer Intervention (Ref: Control Schools) | | | Wave | | | Shorter Intervention x Wave | | | Longer Intervention x Wave | | |
| --- | --- | --- | --- | --- | --- | --- | --- | --- | --- | --- | --- | --- | --- | --- | --- |
| *Outcomes* | *Estimates* | *CI* | *p* | *Estimates* | *CI* | *p* | *Estimates* | *CI* | *p* | *Estimates* | *CI* | *p* | *Estimates* | *CI* | *p* |
| Life skills | -0.77 | -1.09 – -0.45 | **<0.001** | -0.36 | -0.68 – -0.04 | **0.026** | 0.13 | 0.01 – 0.25 | **0.036** | 0.70 | 0.49 – 0.91 | **<0.001** | 0.21 | 0.001 – 0.43 | **0.049** |
| Resilience | -0.08 | -0.43 – 0.27 | 0.643 | 0.33 | -0.02 – 0.67 | 0.063 | -0.07 | -0.20 – 0.06 | 0.306 | 0.10 | -0.14 – 0.33 | 0.421 | -0.16 | -0.39 – 0.07 | 0.176 |
| Self-Efficacy | -0.86 | -1.18 – -0.53 | **<0.001** | -0.80 | -1.12 – -0.48 | **<0.001** | 0.14 | 0.02 – 0.26 | **0.021** | 0.64 | 0.43 – 0.86 | **<0.001** | 0.46 | 0.24 – 0.67 | **<0.001** |
| Stigma | 1.15 | 0.83 – 1.48 | **<0.001** | 0.16 | -0.16 – 0.49 | 0.320 | -0.43 | -0.55 – -0.30 | **<0.001** | -0.68 | -0.90 – -0.46 | **<0.001** | -0.08 | -0.29 – 0.14 | 0.486 |
| Discrimination | 0.69 | 0.34 – 1.03 | **<0.001** | 0.85 | 0.51 – 1.19 | **<0.001** | -0.35 | -0.48 – -0.23 | **<0.001** | -0.30 | -0.53 – -0.07 | **0.011** | -0.50 | -0.73 – -0.27 | **<0.001** |
| Depression | 0.70 | 0.34 – 1.05 | **<0.001** | -0.06 | -0.41 – 0.29 | 0.737 | -0.12 | -0.25 – 0.01 | 0.073 | -0.35 | -0.59 – -0.11 | **0.004** | 0.08 | -0.15 – 0.31 | 0.508 |
| Anxiety | 0.80 | 0.46 – 1.15 | **<0.001** | 0.11 | -0.23 – 0.44 | 0.532 | -0.33 | -0.46 – -0.20 | **<0.001** | -0.37 | -0.60 – -0.15 | **0.001** | -0.03 | -0.26 – 0.19 | 0.772 |
| Reading/general knowledge | -0.23 | -0.49 – 0.04 | 0.091 | -0.73 | -0.99 – -0.47 | **<0.001** | 0.20 | 0.10 – 0.29 | **<0.001** | 0.29 | 0.13 – 0.46 | **0.001** | 0.48 | 0.32 – 0.65 | **<0.001** |
| Numeracy | -0.28 | -0.56 – 0.00 | 0.051 | -0.78 | -1.05 – -0.50 | **<0.001** | 0.29 | 0.19 – 0.39 | **<0.001** | 0.24 | 0.06 – 0.42 | **0.009** | 0.56 | 0.39 – 0.74 | **<0.001** |
| Arithmetic problem solving | -0.33 | -0.66 – -0.01 | **0.041** | -0.35 | -0.66 – -0.03 | **0.032** | -0.09 | -0.21 – 0.03 | 0.142 | 0.37 | 0.16 – 0.58 | **0.001** | 0.25 | 0.04 – 0.46 | **0.018** |

Note: Standard errors are clustered at the school level. Age, gender, ethnicity, assets, and livestock were used as control variables.

Table S8a. Effects of intervention on child mental well-being outcomes, school-based stigma and discrimination, depression, anxiety, and academic outcomes, including covariates in Ghazni

|  | Intervention | | | Wave | | | Intervention × Wave | | |
| --- | --- | --- | --- | --- | --- | --- | --- | --- | --- |
| *Outcomes* | *Estimates* | *CI* | *p* | *Estimates* | *CI* | *p* | *Estimates* | *CI* | *p* |
| Life skills | 0.68 | 0.51 – 0.86 | **<0.001** | 0.28 | 0.20 – 0.36 | **<0.001** | -0.38 | -0.49 – -0.27 | **<0.001** |
| Resilience | 0.08 | -0.04 – 0.21 | 0.198 | 0.23 | 0.17 – 0.28 | **<0.001** | -0.07 | -0.15 – 0.01 | 0.081 |
| Self-Efficacy | 0.51 | 0.30 – 0.71 | **<0.001** | 0.25 | 0.16 – 0.34 | **<0.001** | -0.27 | -0.41 – -0.14 | **<0.001** |
| Stigma | -0.60 | -0.88 – -0.32 | **<0.001** | -0.45 | -0.58 – -0.32 | **<0.001** | 0.37 | 0.19 – 0.55 | **<0.001** |
| Discrimination | 0.18 | -0.10 – 0.45 | 0.211 | -0.11 | -0.23 – 0.02 | 0.096 | -0.24 | -0.41 – -0.06 | **0.009** |
| Depression | -0.53 | -0.78 – -0.27 | **<0.001** | -0.54 | -0.66 – -0.43 | **<0.001** | 0.26 | 0.10 – 0.42 | **0.002** |
| Anxiety | -0.53 | -0.79 – -0.28 | **<0.001** | -0.55 | -0.66 – -0.43 | **<0.001** | 0.26 | 0.10 – 0.43 | **0.002** |
| Reading/general knowledge | 0.03 | -0.15 – 0.21 | 0.740 | 0.24 | 0.17 – 0.31 | **<0.001** | -0.05 | -0.15 – 0.05 | 0.311 |
| Numeracy | 0.23 | 0.05 – 0.40 | **0.010** | 0.34 | 0.27 – 0.41 | **<0.001** | -0.09 | -0.19 – 0.01 | 0.068 |
| Arithmetic problem solving | 0.49 | 0.29 – 0.69 | **<0.001** | 0.32 | 0.24 – 0.41 | **<0.001** | -0.24 | -0.36 – -0.12 | **<0.001** |

Note: Standard errors are clustered at the school level. Age, gender, ethnicity, assets, and livestock were used as control variables.

Table S8b. Effects of intervention length on child mental well-being outcomes, school-based stigma and discrimination, depression, anxiety, and academic outcomes, including covariates in Ghazni

|  | Shorter Intervention (Ref: Control Schools) | | | | Longer Intervention (Ref: Control Schools) | | | | | | Wave | | | | | | Shorter Intervention x Wave | | | | | | | Longer Intervention x Wave | | | | | | |
| --- | --- | --- | --- | --- | --- | --- | --- | --- | --- | --- | --- | --- | --- | --- | --- | --- | --- | --- | --- | --- | --- | --- | --- | --- | --- | --- | --- | --- | --- | --- |
| *Outcomes* | *Estimates* | *CI* | *p* | | *Estimates* | | *CI* | | *p* | | *Estimates* | | *CI* | *p* | | *Estimates* | | | *CI* | | *p* | | *Estimates* | | | *CI* | | *p* | |  |
| Life skills | 0.54 | 0.28 – 0.80 | | **<0.001** | | 0.74 | | 0.55 – 0.94 | | **<0.001** | | 0.28 | 0.20 – 0.36 | | **<0.001** | | | -0.22 | | -0.38 – -0.06 | | **0.009** | | | -0.45 | | -0.57 – -0.33 | | **<0.001** |  |
| Resilience | -0.15 | -0.34 – 0.03 | | 0.105 | | 0.17 | | 0.03 – 0.31 | | **0.017** | | 0.22 | 0.17 – 0.28 | | **<0.001** | | | 0.07 | | -0.05 –0.19 | | 0.273 | | | -0.12 | | -0.21 –-0.03 | | **0.006** |  |
| Self-Efficacy | 0.45 | 0.15 – 0.76 | | **0.004** | | 0.52 | | 0.29 – 0.74 | | **<0.001** | | 0.25 | 0.16 – 0.34 | | **<0.001** | | | -0.21 | | -0.40 –-0.01 | | **0.038** | | | -0.29 | | -0.44 – -0.15 | | **<0.001** |  |
| Stigma | -0.70 | -1.12 – -0.29 | | **0.001** | | -0.55 | | -0.86 – -0.24 | | **0.001** | | -0.44 | -0.57 – -0.32 | | **<0.001** | | | 0.39 | | 0.12 – 0.65 | | **0.004** | | | 0.36 | | 0.17 – 0.56 | | **<0.001** |  |
| Discrimination | -0.03 | -0.43 – 0.38 | | 0.899 | | 0.23 | | -0.08 – 0.53 | | 0.141 | | -0.11 | -0.23 – 0.01 | | 0.080 | | | -0.09 | | -0.35 – 0.17 | | 0.521 | | | -0.28 | | -0.48 –-0.09 | | **0.004** |  |
| Depression | 0.15 | -0.23 – 0.52 | | 0.443 | | -0.80 | | -1.08 – -0.52 | | **<0.001** | | -0.54 | -0.65 – -0.42 | | **<0.001** | | | -0.14 | | -0.37 – 0.10 | | 0.265 | | | 0.41 | | 0.24 – 0.59 | | **<0.001** |  |
| Anxiety | 0.18 | -0.20 – 0.56 | | 0.352 | | -0.82 | | -1.10 – -0.54 | | **<0.001** | | -0.54 | -0.66 – -0.43 | | **<0.001** | | | -0.15 | | -0.39 – 0.09 | | 0.219 | | | 0.43 | | 0.25 – 0.61 | | **<0.001** |  |
| Reading/general knowledge | -0.09 | -0.35 – 0.18 | | 0.514 | | 0.09 | | -0.11 – 0.28 | | 0.385 | | 0.24 | 0.17 – 0.31 | | **<0.001** | | | 0.14 | | -0.01 – 0.29 | | 0.059 | | | -0.13 | | -0.24 – -0.02 | | **0.016** |  |
| Numeracy | 0.03 | -0.22 – 0.29 | | 0.808 | | 0.31 | | 0.12 – 0.50 | | **0.001** | | 0.34 | 0.27 – 0.41 | | **<0.001** | | | 0.18 | | 0.03 – 0.32 | | **0.020** | | | -0.21 | | -0.31 – -0.10 | | **<0.001** |  |
| Arithmetic problem solving | 0.52 | 0.22 – 0.81 | | **0.001** | | 0.48 | | 0.26 – 0.70 | | **<0.001** | | 0.32 | 0.24 – 0.41 | | **<0.001** | | | -0.12 | | -0.30 – 0.06 | | 0.201 | | | -0.29 | | -0.43 – -0.16 | | **<0.001** |  |

Note: Standard errors are clustered at the school level. Age, gender, ethnicity, assets, and livestock were used as control variables.

Table S9a. Effects of intervention on child mental well-being outcomes, school-based stigma and discrimination, depression, anxiety, and academic outcomes including covariates in Jaghori

|  | Intervention | | | Wave | | | Intervention × Wave | | |
| --- | --- | --- | --- | --- | --- | --- | --- | --- | --- |
| *Outcomes* | *Estimates* | *CI* | *p* | *Estimates* | *CI* | *p* | *Estimates* | *CI* | *p* |
| Life skills | 0.55 | 0.20 – 0.90 | **0.002** | 0.12 | -0.04 – 0.27 | 0.144 | -0.27 | -0.49 – -0.05 | **0.017** |
| Resilience | 0.03 | -0.33 – 0.38 | 0.884 | -0.23 | -0.39 – -0.07 | **0.005** | -0.04 | -0.27 – 0.18 | 0.702 |
| Self-Efficacy | 0.49 | 0.15 – 0.84 | **0.005** | -0.08 | -0.24 – 0.07 | 0.282 | -0.24 | -0.46 – -0.02 | **0.030** |
| Stigma | 0.82 | 0.48 – 1.16 | **<0.001** | -0.37 | -0.52 – -0.22 | **<0.001** | -0.33 | -0.54 – -0.11 | **0.003** |
| Discrimination | -0.08 | -0.43 – 0.28 | 0.673 | -0.34 | -0.49 – -0.18 | **<0.001** | 0.31 | 0.08 – 0.53 | **0.007** |
| Depression | 0.04 | -0.31 – 0.38 | 0.844 | -0.17 | -0.33 – -0.01 | **0.032** | -0.09 | -0.31 – 0.14 | 0.447 |
| Anxiety | -0.06 | -0.40 – 0.29 | 0.745 | -0.35 | -0.50 – -0.20 | **<0.001** | -0.04 | -0.26 – 0.18 | 0.735 |
| Reading/general knowledge | -0.22 | -0.46 – 0.03 | 0.085 | 0.04 | -0.05 – 0.14 | 0.373 | 0.09 | -0.05 – 0.22 | 0.208 |
| Numeracy | -0.15 | -0.43 – 0.14 | 0.305 | -0.03 | -0.15 – 0.09 | 0.654 | 0.08 | -0.09 – 0.24 | 0.381 |
| Arithmetic problem solving | -0.22 | -0.52 – 0.07 | 0.139 | -0.02 | -0.15 – 0.11 | 0.745 | 0.10 | -0.08 – 0.28 | 0.286 |

Note: Standard errors are clustered at the school level. Age, gender, ethnicity, assets, and livestock were used as control variables.

Table S9b. Effects of intervention length on child mental well-being outcomes, school-based stigma and discrimination, depression, anxiety, and academic outcomes, including covariates in Jaghori

|  | Shorter Intervention (Ref: Control Schools) | | | | | | Longer Intervention (Ref: Control Schools) | | | | | | | | | | Wave | | | | | | | | Shorter Intervention x Wave | | | | | | | | | | Longer Intervention x Wave | | | | | | | | | |  |  |
| --- | --- | --- | --- | --- | --- | --- | --- | --- | --- | --- | --- | --- | --- | --- | --- | --- | --- | --- | --- | --- | --- | --- | --- | --- | --- | --- | --- | --- | --- | --- | --- | --- | --- | --- | --- | --- | --- | --- | --- | --- | --- | --- | --- | --- | --- | --- |
| *Outcomes* | *Estimates* | *CI* | *p* | | | | *Estimates* | | | *CI* | | *p* | | | | | *Estimates* | | | *CI* | | *p* | | | *Estimates* | | | *CI* | | *p* | | | | | *Estimates* | | | *CI* | | | | *p* | | | | |
| Life skills | 0.46 | 0.09 – 0.83 | | | **0.014** | | | 0.94 | | | 0.27 – 1.60 | | | **0.006** | | 0.11 | | | -0.04 – 0.27 | | | | 0.154 | | | -0.22 | | | -0.45 – 0.02 | | | 0.067 | | -0.51 | | | -0.94 – -0.08 | | | | **0.021** | | | | |  |
| Resilience | 0.08 | -0.30 – 0.45 | 0.688 | | | | | -0.33 | | | -1.00 – 0.35 | | | 0.341 | | -0.23 | | | -0.39 – -0.07 | | | | **0.004** | | | -0.10 | | | -0.34 – 0.13 | | | 0.385 | | 0.28 | | | -0.15 – 0.72 | | | | 0.203 | | | | |  |
| Self-Efficacy | 0.48 | 0.12 – 0.85 | **0.010** | | | | | 0.45 | | | -0.21 – 1.11 | | | 0.177 | | -0.09 | | | -0.24 – 0.07 | | | | 0.261 | | | -0.26 | | | -0.49 – -0.03 | | | **0.029** | | -0.15 | | | -0.58 – 0.27 | | | | 0.483 | | | | |  |
| Stigma | 0.84 | 0.48 – 1.20 | **<0.001** | | | | | 0.46 | | | -0.18 – 1.10 | | | 0.161 | | -0.38 | | | -0.53 – -0.23 | | | | **<0.001** | | | -0.39 | | | -0.61 – -0.16 | | | **0.001** | | 0.06 | | | -0.36 – 0.48 | | | | 0.773 | | | | |  |
| Discrimination | -0.05 | -0.42 – 0.32 | 0.787 | | | | | -0.22 | | | -0.90 – 0.45 | | | 0.513 | | -0.34 | | | -0.50 – -0.18 | | | | **<0.001** | | | 0.28 | | | 0.04 – 0.52 | | | **0.020** | | 0.45 | | | 0.01 – 0.89 | | | | **0.044** | | | | |  |
| Depression | -0.03 | -0.40 – 0.34 | 0.882 | | | | | 0.15 | | | -0.52 – 0.81 | | | 0.664 | | -0.18 | | | -0.33 – -0.02 | | | | **0.026** | | | -0.09 | | | -0.32 – 0.14 | | | 0.445 | | -0.02 | | | -0.45 – 0.42 | | | | 0.945 | | | | |  |
| Anxiety | -0.15 | -0.52 – 0.21 | | 0.417 | | 0.28 | | | -0.38 – 0.93 | | | | 0.411 | | -0.35 | | | -0.51 – -0.20 | | | **<0.001** | | | -0.02 | | | -0.26 – 0.21 | | | | 0.839 | | -0.10 | | | -0.53 – 0.33 | | | | 0.654 | | | |  |  |  |
| Reading/general knowledge | -0.24 | -0.50 – 0.02 | | 0.072 | | -0.20 | | | -0.66 – 0.26 | | | | 0.388 | | 0.04 | | | -0.06 – 0.14 | | | 0.404 | | | 0.08 | | | -0.06 – 0.22 | | | | 0.292 | | 0.15 | | | -0.11 – 0.41 | | | | 0.263 | | | |  |  |  |
| Numeracy | -0.19 | -0.49 – 0.11 | | 0.209 | | 0.01 | | | -0.52 – 0.54 | | | | 0.963 | | -0.03 | | | -0.15 – 0.09 | | | 0.637 | | | 0.08 | | | -0.09 – 0.26 | | | | 0.355 | | 0.04 | | | -0.29 – 0.37 | | | 0.828 | | | |  |  |  |  |
| Arithmetic problem solving | -0.27 | -0.59 – 0.04 | | 0.087 | | -0.08 | | | -0.63 – 0.48 | | | | 0.792 | | -0.02 | | | -0.15 – 0.10 | | | 0.719 | | | 0.12 | | | -0.07 – 0.31 | | | | 0.221 | | 0.02 | | | -0.33 – 0.38 | | | 0.896 | | | |  |  |  |  |

Note: Standard errors are clustered at the school level. Age, gender, ethnicity, assets, and livestock were used as control variables.

Table S10a. Effects of intervention on child mental well-being outcomes, school-based stigma and discrimination, depression, anxiety, and academic outcomes, including covariates in Takhar

|  | Intervention | | | Wave | | | Intervention × Wave | | |
| --- | --- | --- | --- | --- | --- | --- | --- | --- | --- |
| *Outcomes* | *Estimates* | *CI* | *p* | *Estimates* | *CI* | *p* | *Estimates* | *CI* | *p* |
| Life skills | 0.48 | 0.24 – 0.72 | **<0.001** | -0.13 | -0.23 – -0.02 | **0.016** | -0.30 | -0.45 – -0.15 | **<0.001** |
| Resilience | 0.17 | -0.07 – 0.42 | 0.170 | 0.13 | 0.02 – 0.24 | **0.020** | -0.05 | -0.21 – 0.11 | 0.528 |
| Self-Efficacy | 0.20 | -0.04 – 0.44 | 0.111 | -0.18 | -0.28 – -0.08 | **0.001** | -0.16 | -0.30 – -0.01 | **0.040** |
| Stigma | -0.73 | -0.98 – -0.48 | **<0.001** | 0.35 | 0.24 – 0.46 | **<0.001** | 0.49 | 0.33 – 0.65 | **<0.001** |
| Discrimination | 0.46 | 0.19 – 0.72 | **0.001** | 0.04 | -0.08 – 0.16 | 0.497 | -0.35 | -0.52 – -0.19 | **<0.001** |
| Depression | 0.46 | 0.19 – 0.72 | **0.001** | -0.12 | -0.24 – -0.01 | **0.038** | -0.28 | -0.44 – -0.11 | **0.001** |
| Anxiety | 0.25 | -0.01 – 0.52 | 0.062 | 0.27 | 0.15 – 0.38 | **<0.001** | -0.09 | -0.25 – 0.08 | 0.320 |
| Reading/general knowledge | -0.59 | -0.81 – -0.37 | **<0.001** | 0.23 | 0.14 – 0.32 | **<0.001** | 0.45 | 0.32 – 0.57 | **<0.001** |
| Numeracy | -0.63 | -0.84 – -0.41 | **<0.001** | 0.16 | 0.06 – 0.25 | **0.001** | 0.41 | 0.28 – 0.55 | **<0.001** |
| Arithmetic problem solving | -0.46 | -0.71 – -0.22 | **<0.001** | 0.07 | -0.03 – 0.18 | 0.171 | 0.23 | 0.07 – 0.38 | **0.004** |

Note: Standard errors are clustered at the school level. Age, gender, ethnicity, assets, and livestock were used as control variables.

Table S10b. Effects of intervention length on child mental well-being outcomes, school-based stigma and discrimination, depression, anxiety, and academic outcomes, including covariates in Takhar

|  | | | Shorter Intervention (Ref: Control Schools) | | | | | | Longer Intervention (Ref: Control Schools) | | | | | | | Wave | | | | | | Shorter Intervention x Wave | | | | | | | Longer Intervention x Wave | | | | | | |  |  |
| --- | --- | --- | --- | --- | --- | --- | --- | --- | --- | --- | --- | --- | --- | --- | --- | --- | --- | --- | --- | --- | --- | --- | --- | --- | --- | --- | --- | --- | --- | --- | --- | --- | --- | --- | --- | --- | --- |
| *Outcomes* | | *Estimates* | | | *CI* | | *p* | | | *Estimates* | | *CI* | | *p* | | | *Estimates* | | *CI* | *p* | | | *Estimates* | | | *CI* | *p* | | | *Estimates* | | *CI* | | *p* | | |  |
| Life skills | -0.46 | | | -1.06 – 0.13 | | 0.126 | | 0.57 | | | 0.32 – 0.82 | | **<0.001** | | -0.13 | | | -0.23 – -0.03 | | | **0.013** | | | 0.34 | -0.03 – 0.71 | | | 0.069 | | | -0.36 | | -0.51 – -0.21 | | **<0.001** | | |
| Resilience | -0.37 | | | -0.98 – 0.25 | | 0.244 | | 0.20 | | | -0.05 – 0.46 | | 0.121 | | 0.12 | | | 0.01 – 0.23 | | | **0.026** | | | 0.29 | -0.10 – 0.68 | | | 0.142 | | | -0.07 | | -0.23 – 0.09 | | 0.379 | | |
| Self-Efficacy | -0.68 | | | -1.27 – -0.08 | | **0.025** | | 0.27 | | | 0.03 – 0.52 | | **0.029** | | -0.18 | | | -0.28 – -0.08 | | | **<0.001** | | | 0.46 | 0.10 – 0.83 | | | **0.013** | | | -0.21 | | -0.36 – -0.06 | | **0.006** | | |
| Stigma | 0.33 | | | -0.29 – 0.95 | | 0.295 | | -0.83 | | | -1.08 – -0.57 | | **<0.001** | | 0.36 | | | 0.25 – 0.46 | | | **<0.001** | | | -0.25 | -0.64 – 0.14 | | | 0.205 | | | 0.56 | | 0.40 – 0.72 | | **<0.001** | | |
| Discrimination | 0.49 | | | -0.18 – 1.15 | | 0.151 | | 0.47 | | | 0.20 – 0.75 | | **0.001** | | 0.04 | | | -0.07 – 0.16 | | | 0.458 | | | -0.42 | -0.83 – 0.00 | | | 0.051 | | | -0.36 | | -0.53 – -0.19 | | **<0.001** | | |
| Depression | -0.03 | | | -0.68 – 0.62 | | 0.927 | | 0.53 | | | 0.26 – 0.80 | | **<0.001** | | -0.12 | | | -0.23 – -0.00 | | | **0.044** | | | 0.11 | -0.30 – 0.53 | | | 0.596 | | | -0.33 | | -0.50 – -0.16 | | **<0.001** | | |
| Anxiety | -0.07 | | | -0.73 – 0.59 | | 0.829 | | 0.29 | | | 0.02 – 0.56 | | **0.037** | | 0.27 | | | 0.15 – 0.38 | | | **<0.001** | | | 0.27 | -0.14 – 0.69 | | | 0.199 | | | -0.12 | | -0.30 – 0.05 | | 0.162 | | |
| Reading/general knowledge | -1.34 | | | -1.87 – -0.81 | | **<0.001** | | -0.55 | | | -0.77 – -0.32 | | **<0.001** | | 0.22 | | | 0.13 – 0.31 | | | **<0.001** | | | 0.53 | 0.21 – 0.86 | | | **0.001** | | | 0.45 | | 0.32 – 0.58 | | **<0.001** | | |
| Numeracy | -1.60 | | | -2.14 – -1.06 | | **<0.001** | | -0.54 | | | -0.76 – -0.32 | | **<0.001** | | 0.16 | | | 0.06 – 0.25 | | | **0.001** | | | 0.77 | 0.44 – 1.10 | | | **<0.001** | | | 0.38 | | 0.25 – 0.52 | | **<0.001** | | |
| Arithmetic problem solving | -1.75 | | | -2.36 – -1.14 | | **<0.001** | | -0.37 | | | -0.62 – -0.12 | | **0.004** | | 0.07 | | | -0.04 – 0.17 | | | 0.197 | | | 0.85 | 0.47 – 1.23 | | | **<0.001** | | | 0.18 | | 0.02 – 0.34 | | **0.024** | | |

Note: Standard errors are clustered at the school level. Age, gender, ethnicity, assets, and livestock were used as control variables.
